# First detection and genetic characterisation of *Enterocytozoon bieneusi* in wild deer in Melbourne’s water catchments in Australia

**DOI:** 10.1186/s13071-017-2577-7

**Published:** 2018-01-03

**Authors:** Yan Zhang, Anson V. Koehler, Tao Wang, Shane R. Haydon, Robin B. Gasser

**Affiliations:** 1 Department of Veterinary Biosciences, Melbourne Veterinary School, Faculty of Veterinary and Agricultural Sciences, The University of Melbourne, Parkville, VIC 3010 Australia; 2 Melbourne Water, Docklands, VIC 3001 Australia

**Keywords:** *Enterocytozoon bieneusi*, Genotypes, Internal transcribed spacer (ITS) of nuclear ribosomal DNA, Prevalence, Sambar deer, Australia

## Abstract

**Background:**

*Enterocytozoon bieneusi* is reported to be a common microsporidian of humans and animals in various countries. However, *E. bieneusi* has yet to be recorded in animals in Australia. Here, we undertook the first molecular epidemiological investigation of *E. bieneusi* in three species of deer (*Cervus elaphus*, *Dama dama* and *Rusa unicolor*) that live in the catchment areas that supply the city of Melbourne with drinking water.

**Methods:**

Genomic DNA was extracted from a total of 610 individual faecal samples from wild deer, including sambar deer (*Rusa unicolor*) (*n* = 516), red deer (*Cervus elaphus*) (*n* = 77) and fallow deer (*Dama dama*) (*n* = 17) from nine catchment areas, and then tested using a nested PCR-based sequencing approach employing internal transcribed spacer (ITS) of nuclear ribosomal DNA as the genetic marker.

**Results:**

*Enterocytozoon bieneusi* was detected in 25 of all 610 (4.1%) samples exclusively in samples from sambar deer. The analysis of ITS sequence data revealed three known (D, J and Type IV) and two new (MWC_d1 and MWC_d2) genotypes of *E. bieneusi*. Although the significance of the latter two new genotypes is presently unknown, phylogenetic analysis of ITS sequence data sets showed that they cluster with genotypes D and Type IV, which have been recorded previously in humans. These findings suggest that sambar deer in the water catchments harbour zoonotic genotypes of *E. bieneusi*.

**Conclusions:**

Further insight into the epidemiology of *E. bieneusi* in wildlife, water and the environment in Australia will be important to have an informed position on the public health significance of microsporidiosis caused by this microbe.

**Electronic supplementary material:**

The online version of this article (10.1186/s13071-017-2577-7) contains supplementary material, which is available to authorized users.

## Background

*Enterocytozoon bieneusi* is a member of the Microsporidia [1–3] and is recognised as a fungus, although its exact classification is currently being discussed [4–8]. More than 1200 microsporidia have been recorded from a range of animals species [9]. Currently, 17 microsporidian species are recognised to infect humans, of which *E. bieneusi* is most commonly found to be pathogenic [10].

*Enterocytozoon bieneusi* was first detected in a Haitian patient with HIV/AIDS who suffered from severe diarrhoea [11]. Numerous published reports (reviewed in [10, 12, 13]) show that this species can infect both immunocompromised and immunocompetent people. *Enterocytozoon bieneusi* is often an opportunistic pathogen in the former group of humans [12, 14–17], typically causing mild to severe, acute or chronic diarrhoea, malabsorption and/or wasting [18–21]. Some individuals infected with *E. bieneusi* do not show clinical signs and thus represent carriers of this microsporidian, with the potential of spreading spores to the environment and/or other people or animals, which is an important epidemiological consideration [1, 22–24]. Humans and animals are usually infected via a faecal-oral transmission route, either through spore-contaminated water [25, 26] or food [27, 28]. Interestingly, *E. bieneusi* has also been detected in the respiratory tracts of AIDS patients; however, whether *E. bieneusi* is capable of airborne transmission requires verification [29–31]. Nonetheless, a study has reported that *E. bieneusi* spores can be found in air and could potentially be inhaled by humans [32].

*Enterocytozoon bieneusi* isolates are usually characterised genetically by sequencing their internal transcribed spacer region (ITS) of nuclear ribosomal DNA. To date, more than 200 distinct genotypes have been reported in the literature (reviewed in [10, 12]). Some of them appear to be host-specific or host-adapted. However, others can be found in different species of animals as well as humans, indicating the zoonotic potential of these *E. bieneusi* genotypes (e.g. D, EbpA, J, K and Type IV; [12]). Thus, the National Institute of Allergy and Infectious Diseases (NIAID) has classified *E. bieneusi* as a Category B Priority Pathogen [33].

Various studies [34–51] have assessed the diversity of genotypes of *E. bieneusi* in a broad range of wild and domestic animal hosts, including mammals (carnivores, lagomorphs, primates, rodents and ungulates) and birds, in as many as 39 countries. However, almost nothing is known about the transmission routes of most genotypes or their zoonotic capacity. Moreover, with the exception of records of *E. bieneusi* in HIV/AIDS patients [14, 52], surprisingly, there is no published record of *E. bieneusi* in any species of animals from Australia. The present study takes a first step to investigating *E. bieneusi* in animals in Australia. Using a molecular approach, we explored the presence and distribution of this microsporidian in wild deer inhabiting catchment areas (managed by the Melbourne Water Corporation, MWC) that supply people in the city of Melbourne and environs with treated (but unfiltered) drinking water.

## Methods

### Melbourne’s water catchments

Ten main water catchment reservoirs (www.melbournewater.com.au) supply drinking water to the people of Melbourne and environs. Approximately 80% of Melbourne’s drinking water is drawn from ‘closed’ catchments in the Yarra Ranges (east of Melbourne). These catchments cover almost 160,000 ha of eucalypt forest. Access by humans and domestic animals is restricted. The remaining 20% of Melbourne’s water comes from ‘open’ catchments, in which some farming and human activities are permitted. All water undergoes treatment, in accordance with national and international guidelines [53, 54]. The nine catchment areas studied here are located north and east of Melbourne, are less than 90 km apart and include: Armstrong (AM) 37°38'S 145°51'E; Cardinia (CA) 37°47'S 145°24'E; Maroondah (MR) 37°38'S 145°33'E; O’Shannassy (OS) 37°40'S 145°48'E; Silvan (SV) 37°50'S 145°25'E; Tarago (TAR) 37°59'S 145°55'E; Thompson (TH) 37°47'S 146°21'E; Upper Yarra (UY) 37°40'S 145°55'E and Yan Yean (YY) 37°33'S 145°08'E [55, 56]. Reservoirs MR, OS, TH and UY are situated in the densely forested Yarra Ranges catchment, whereas the YY reservoir is a much smaller catchment north of Melbourne and surrounded by residential and grazing land. The other reservoirs, including CA and SV, act as storage facilities for the larger catchments, and have eucalypt and/or pine forests. TAR is the one ‘open’ water-supply catchment where farming in the land surrounding the reservoir is permitted. Small areas of grassland abut some water reservoirs, from where faecal samples were collected in this study. Most of the faecal samples from deer collected here originate from catchment areas CA, MR, OS, UY and YY.

### Samples and DNA isolation

A total of 610 faecal samples from wild deer, including sambar deer (*Rusa unicolor*; *n* = 516), red deer (*Cervus elaphus*; *n* = 77) and fallow deer (*Dama dama*; *n* = 17), were collected from nine of Melbourne’s water catchments from June 2009 to March 2017. Specifically, samples were collected from AM (*n* = 12), CA (*n* = 114), MR (*n* = 98), OS (*n* = 112), SV (*n* = 3), TAR (*n* = 16), TH (*n* = 8), UY (*n* = 134) and YY (*n* = 113). Genomic DNA was extracted directly from 0.2 g of each of the 610 faecal samples using the PowerSoil kit (MoBio, Carlsbad, CA, USA), according to the manufacturer’s instructions, and stored at -20 °C. This kit was used, because it is highly effective at removing components from faecal samples that are inhibitory to PCR [56]. Scats were initially identified using a field guide [57], and host identity was unequivocally confirmed by testing faecal DNA using a nested PCR-based sequencing approach employing the mitochondrial cytochrome *b* gene (cf. [58]).

### Nested PCR-based sequencing of *E. bieneusi* ITS

From individual faecal DNA samples, the internal transcribed spacer (ITS) of nuclear ribosomal DNA of *E. bieneusi* was specifically amplified by PCR using degenerate primers originally designed and evaluated by Katzwinkel-Wladarsch et al. [59]. In the first PCR round, primers MSP-1 (forward: 5′-TGA ATG KGT CCC TGT-3′) and MSP-2B (reverse: 5′-GTT CAT TCG CAC TAC T-3′) were used to amplify 601 bp of ITS plus flanking gene sequences. In the second round, primers MSP-3 (forward: 5′-GGA ATT CAC ACC GCC CGT CRY TAT-3′) and MSP-4B (reverse: 5′-CCA AGC TTA TGC TTA AGT CCA GGG AG-3′) were employed to amplify a product of 535 bp containing 130 bp of the 3′-end of the small subunit (*SSU*) of the nuclear rRNA gene, 243 bp of the ITS and 162 bp of the 5′-region of the large subunit (*LSU*) rRNA gene.

Nested PCR (in 50 μl) was conducted in a standard buffer containing 3.0 μM MgCl_2,_ 0.4 mM dNTPs, 50 pmol of each primer, 1.25 U of Mango*Taq* polymerase (Bioline, London, UK) and DNA template - except for the negative (no-template) control. The cycling conditions for both primary and secondary (nested) PCRs were: 94 °C for 5 min (initial denaturation), followed by 35 cycles of 94 °C for 45 s (denaturation), 54 °C for 45 s (annealing) and 72 °C for 60 s (extension), followed by 72 °C for 10 min (final extension). Known test-positive, test-negative and no-template controls were included in each PCR run.

PCR products were examined on ethidium bromide-stained 1.5% agarose gels using TBE (65 mM Tris-HCl, 27 mM boric acid, 1 mM EDTA, pH 9; Bio-Rad, Hercules, CA, USA) as the buffer and using 100 bp DNA ladder (Promega, Madison, WI, USA) as a size marker. Subsequently, secondary amplicons were individually treated with ExoSAP-IT (Affymetrix, Santa Clara, CA, USA) according to the manufacturer’s instructions and directly sequenced (BigDye Terminator v.3.1 chemistry, Applied Biosystems, Foster City, CA, USA) using primers MSP-3 and MSP-4B in separate reactions. ITS sequences were aligned and analysed using the program Geneious v.10 [60] and compared with sequences from the GenBank database (Additional file 1: Table S1). *Enterocytozoon bieneusi* sequence types or genotypes were named according to a recent recommendation (i.e. GenBank accession number for the sequence, followed by a brief description) [13, 61]. Sequences from the present study were submitted to the GenBank database (NCBI) and assigned specific accession numbers (MF496203 and MF496204; MF693831–MF693833).

### Phylogenetic analysis

Bayesian inference (BI) and Monte Carlo Markov Chain (MCMC) analysis in MrBayes v.3.2.3 [62] were used for phylogenetic analysis. Akaike Information Criteria (AIC) test in jModeltest v.2.1.7 [63] was used to evaluate the likelihood parameters set for BI analysis. Posterior probability (pp) values were calculated by running 2,000,000 generations with four simultaneous tree-building chains, with trees being saved every 100th generation. A 50% majority rule consensus tree for each analysis was constructed based on the final 75% of trees generated by BI. In the phylogenetic tree, *E. bieneusi* clades and subclades were assigned using the classification system first proposed by Drosten et al. [51].

## Results

*Enterocytozoon bieneusi* DNA was detected in 25 of 610 (4.1%) faecal samples from wild deer by nested PCR-based sequencing of ITS and exclusively in sambar deer in 5 of 9 water catchment areas; the highest prevalence (9.0%; 12 of 134 samples) was recorded in catchment UY compared with 0–5.3% in the 8 other catchments (Table 1). Test-positive samples were found in all seasons, with the highest prevalence (6.2%; 8/130) of *E. bieneusi* in spring (Table 2); prevalences varied from 1.5–16.7% among years (Table 3).

**Table 1:** The numbers of faecal samples tested for *Enterocytozoon bieneusi* by nested PCR, and individual prevalences of this microsporidian in the nine of Melbourne’s water catchments

Catchment	Total no. of samples tested	No. of test-positive samples	Prevalence (%)
Upper Yarra (UY)	134	12	9.0
Cardinia (CA)	114	6	5.3
O’Shannassay (OS)	112	4	3.6
Maroondah (MR)	98	2	2.0
Yan Yean (YY)	113	1	0.9
Armstrong (AM)	12	0	0
Silvan (SV)	3	0	0
Tarago (TAR)	16	0	0
Thompson (TH)	8	0	0
Total	610	25	4.1

**Table 2:** Seasonal prevalences of *Enterocytozoon bieneusi* in sambar deer in nine of Melbourne’s water catchments

Season	Total no. of samples tested	No. of test-positive samples	Prevalence (%)
Spring	130	8	6.2
Summer	97	2	2.1
Autumn	163	8	5.0
Winter	220	7	3.2

**Table 3:** Annual prevalences of *Enterocytozoon bieneusi* in Sambar deer in nine of Melbourne’s water catchment areas

Year	Total no. of samples tested	No. of test-positive samples	Prevalence (%)
2009	57	1	1.8
2010	60	3	5.0
2011	67	1	1.5
2012	54	2	3.7
2013	67	2	3.0
2014	118	10	8.5
2015	67	2	3.0
2016	114	3	2.6
2017	6	1	16.7

The sequencing of all 25 ITS amplicons (243 bp) and comparisons with reference sequences in the GenBank database identified five distinct sequence types (genotypes). Of the 25 ITS sequences, 3, 1 and 1 were identical to those representing genotypes D, J and Type IV (accession numbers AF101200, AF135837 and AF242478), respectively. In addition, 19 and one ITS sequences represented two novel genotypes, designated MWC_d1 and MWC_d2, respectively, which differed at one or two of 243 nucleotide positions (0.41 to 0.82% difference) from the sequence with accession number KF383397 (*E. bieneusi* “Wildboar2”). Specifically, MWC_d1 was the dominant genotype detected in 4, 2, 2 and 11 samples from catchments CA, OS, MR and UY, respectively (Table 4).

**Table 4:** Genotypes of *Enterocytozoon bieneusi* characterised by nested PCR-based sequencing of the internal transcribed spacer (ITS) region of the nuclear ribosomal DNA from 25 individual faecal samples from sambar deer collected (in different seasons and years) from five of Melbourne’s water catchment areas

Genotype designation^a^	GenBank ID	Sample code	Catchment area	Season	Year
D	MF693831	C1508	Cardinia	Spring	2010
D	MF693831	UY1357	Upper Yarra	Winter	2010
D	MF693831	YY4416	Yan Yean	Autumn	2014
J	MF693833	OS5279	O’Shannassay	Spring	2014
Type IV	MF693832	OS1917	O’Shannassay	Winter	2011
MWC_d1 *	MF496204	C1505	Cardinia	Spring	2010
MWC_d1 *	MF496204	C4255	Cardinia	Summer	2014
MWC_d1 *	MF496204	C3645	Cardinia	Winter	2013
MWC_d1 *	MF496204	C4857	Cardinia	Winter	2014
MWC_d1 *	MF496204	MR7507	Maroondah	Autumn	2017
MWC_d1 *	MF496204	MR240	Maroondah	Winter	2009
MWC_d1 *	MF496204	OS4550	O’Shannassay	Autumn	2014
MWC_d1 *	MF496204	OS7320	O’Shannassay	Spring	2016
MWC_d1 *	MF496204	UY4661	Upper Yarra	Autumn	2014
MWC_d1 *	MF496204	UY4607	Upper Yarra	Autumn	2014
MWC_d1 *	MF496204	UY4646	Upper Yarra	Autumn	2014
MWC_d1 *	MF496204	UY4614	Upper Yarra	Autumn	2014
MWC_d1 *	MF496204	UY4624	Upper Yarra	Autumn	2014
MWC_d1 *	MF496204	UY4067	Upper Yarra	Spring	2013
MWC_d1 *	MF496204	UY6189	Upper Yarra	Spring	2015
MWC_d1 *	MF496204	UY6181	Upper Yarra	Spring	2015
MWC_d1 *	MF496204	UY6540	Upper Yarra	Summer	2016
MWC_d1 *	MF496204	UY2942	Upper Yarra	Winter	2012
MWC_d1 *	MF496204	UY6973	Upper Yarra	Winter	2016
MWC_d2 *	MF496203	C3263	Cardinia	Spring	2012

^a^Novel genotype is indicated with an asterisk

**Fig. 1:**
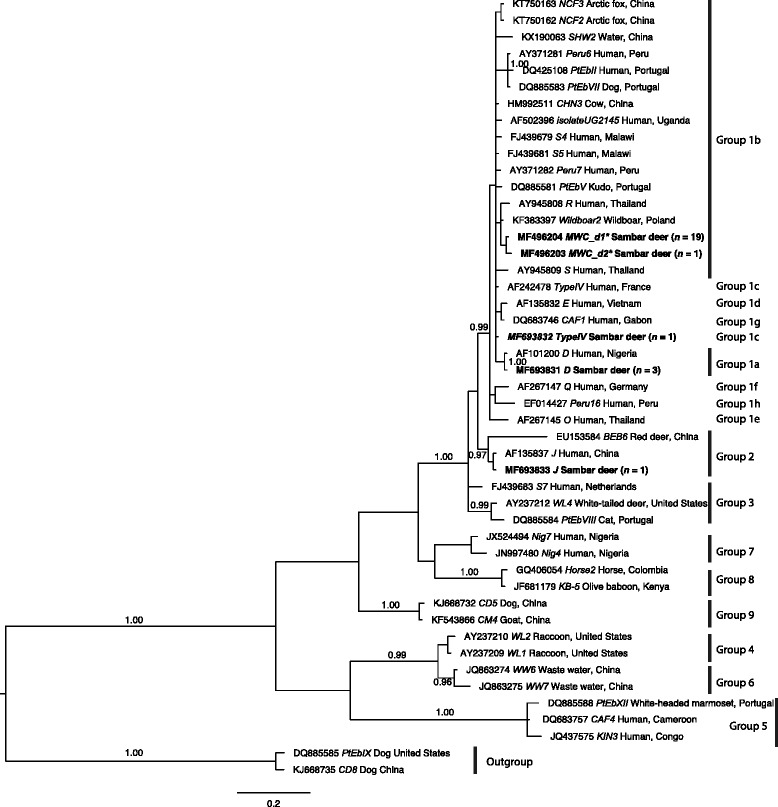
Relationships among genotypes of *Enterocytozoon bieneusi* recorded in sambar deer in this study inferred from phylogenetic analysis of sequence data for the internal transcribed spacer (ITS) of nuclear ribosomal DNA by Bayesian inference (BI). Sequences from a range of distinct *E. bieneusi* genotypes from published papers were included for comparison in the analysis (see Additional file 3: Table S3; [33, 48–50]). Statistically significant posterior probabilities (pp) are indicated on branches. Individual GenBank accession numbers precede genotype designation (in italics), followed by sample and locality descriptions. *Enterocytozoon bieneusi* genotypes identified and characterised from faecal DNA samples in the present study are indicated in bold-type. Clades were assigned group names based on the classification system established by Drosten et al. [51], with Group 1 being further subdivided into Subgroups 1a to 1 h. The scale-bar represents the number of substitutions per site. The *E. bieneusi* genotypes PtEbIX (DQ85585) and CD8 (KJ668735) from dogs were used as outgroups. An asterisk (*) indicates a novel genotype characterised in the present study

The ITS sequence data for the five distinct genotypes (representing 25 samples) were included in a phylogenetic analysis, together with sequences representing nine established groups of *E. bieneusi* (Fig. 1). The major clades were well-supported (pp = 0.96 to 01.00), but there was limited support for some clades within Group 1. Phylogenetic analysis showed that genotypes D and Type IV as well as the two novel genotypes all belonged to Group 1 (pp = 0.99). The novel genotypes clustered with members representing Group 1b without statistical support (pp = 0.90; not shown) and genotype J clustered with representatives of Group 2 (pp = 0.97).

## Discussion

This is the first molecular investigation of *E. bieneusi* in animals in Australia. Here, we investigated the occurrence and prevalence of *E. bieneusi* in faecal samples from three species of deer (*R. unicolor*, *C. elaphus* and *D. dama*) in nine of Melbourne’s water catchment areas. *Enterocytozoon bieneusi* was recorded exclusively in *R. unicolor* (sambar deer) and was detected in 4.1% (25/610) of this species in five of the water catchment areas, with the highest prevalence (12 of 134 samples; 9.0%) in catchment area UY, followed by CA (5.3%), OS (3.6%), MR (2.0%) and YY (0.9%); there was no evidence of this microsporidian being present in deer in catchments AM, SV, TAR and TH. *Enterocytozoon bieneusi* was detected in sambar deer in all seasons, with the highest prevalence (6.2%; 8/130) in spring.

As environmental temperature, ultraviolet (UV) radiation and humidity are key factors that determine the survival of microsporidial spores [64–67], it is hypothesised that high temperature and UV radiation as well as limited rainfall limit the survival of *E. bieneusi* spores during summer, reducing the likelihood of transmission in this season, whereas low to moderate temperatures and UV levels as well as increased rainfall promote spore survival and transmission in spring, autumn and winter. This proposal is supported by some evidence from previous studies showing that the low prevalence of *E. bieneusi* in catchment areas CA and UY in summer (2.1%; 2/97) compared with other seasons was likely due to high environmental temperature [64–66] and UV exposure [67, 68] in this season. In the present study, the prevalence of *E. bieneusi* varied from 1.5% (1/67) in 2011 to 16.7% (1/6) in 2017, which might relate to variability in the aforementioned environmental factors (temperature, UV and humidity) and/or the sizes of deer populations in individual catchments, the health status of deer and aspects relating to wildlife management.

Current information about *E. bieneusi* of wild deer is limited to two published papers. The first is that of Guo et al. [69], who detected *E. bieneusi* in 12.2% (6/49) of wild *Odocoileus virginianus* (white-tailed deer) in the State of New York, USA. The second is that of Santín & Fayer [36], who detected this microporidian in 32.5% (26/80) of the same deer species in Maryland, USA. Nonetheless, there are some studies of *E. bieneusi* in farmed deer from China, which involve *Cervus nippon* (sika deer), *C. elaphus* (red deer) and *Elaphurus davidianus* (Père David’s deer) [36, 40, 69–73] (see Additional file 2: Table S2). As expected, the prevalence of *E. bieneusi* varied considerably (from 7.1 to 44.1%) among different farms, which likely relates to factors such as population size of deer and density; host species, age and gender; health status and immunity of the deer; management and environmental factors - season, temperature, sunlight and humidity - and stress, some of which may exert a combined effect on the survival of microsporidian species, and its transmission and prevalence. Future research might focus on long-term molecular epidemiological studies of *E. bieneusi* in different species of wild and domestic deer of differing ages and health status and in different countries to establish which environmental, climatic and/or management factors mostly influence prevalence and intensity of infection of *E. bieneusi* and the transmission of this microbe.

The present genetic characterisation of *E. bieneusi* from sambar deer revealed three known genotypes (D, J and Type IV; cf. [36, 40, 71, 73]) and two novel genotypes (MWC_d1 and MWC_d2). MWC_d1 was the predominant genotype (76%; 19/25), followed by genotypes D (12%; 3/25), J (4%; 1/25), MWC_d2 (4%; 1/25) and Type IV (4%; 1/25) (i.e. J, MWC_d2 and Type IV have the same prevalence) (Table 4). Although genotypes D, J and Type IV have been detected previously in 55, 18 and 16 different species of vertebrates (see Additional file 3: Table S3) [43, 74–76], respectively, these genotypes are recorded here for the first time in sambar deer. Interestingly, the relatively broad host ranges apparent for these three genotypes (see Additional file 3: Table S3) suggest their potential to spread among different species of animals. Moreover, the presence of genotypes D, J and Type IV in humans suggests that the animal species that harbour these genotypes represent important reservoirs for the transmission of microsporidiosis to humans. Thus, the finding of genotypes D, J and Type IV of sambar deer signals that they might be transmissible to humans. By contrast, the zoonotic potential of the two novel genotypes (MWC_d1 and MWC_d2) of *E. bieneusi* is presently unknown and should be explored further (indirectly) by studying *E. bieneusi* in humans in Melbourne, Australia, who consume unfiltered drinking water originating from Melbourne water catchments.

Sambar deer were introduced to Australia in the 1860s from the Philippines and Sri Lanka [77]. It is possible that *E. bieneusi* originated from these countries. However, it is also possible that sambar deer acquired infection following introduction into Australia. The origin cannot yet be inferred due to the absence of data for *E. bieneusi* in sambar deer and other animals in those two countries. Further studies would be needed to attempt to establish the origin of *E. bieneusi*. The transmission of *E. bieneusi* has been proposed to occur mainly through contaminated food, and drinking, recreational and waste waters [25–27, 78]. All three known genotypes (D, J and Type IV) identified here in sambar deer have also been detected previously in water in China, Ireland, Nigeria, Spain and Tunisia [25, 26, 79–85] (see Additional file 3: Table S3), indicating that *E. bieneusi*-contaminated water might be a key source for transmission of microsporidiosis to various species of animals including humans. This information is a stimulus to explore whether *E. bieneusi* is present in Melbourne’s water.

Phylogenetic analysis of ITS gene sequence data sets have shown previously that genotype J belongs to a ‘cattle-specific’ assemblage (called Group 2; [86]), whereas genotypes D, MWC_d1, MWC_d2 and Type IV identified in the present study are inferred to be in the ‘zoonotic’ assemblage (called Group 1) (Fig. 1). Therefore, it would be interesting to extend studies of *E. bieneusi* to humans in Melbourne and environs, and other species of animals in Melbourne’s water catchments, but perhaps, more importantly, to investigate the presence of *E. bieneusi* in catchment-source and drinking water in Melbourne, Australia. Also, further studies could explore other genetic markers for genotypic characterization of *E. bieneusi* to establish whether a genotype defined by the ITS region is in accord with assignment using a small set of other genetic markers, or a large panel of, for example, single copy genes from the *E. bieneusi* genome.

Since *E. bieneusi* can cause acute or chronic enteritis and sometimes systemic disease [18–21], this microsporidian species has been classified as a Category B Priority Pathogen by the National Institute of Allergy and Infectious Diseases (NIAID) [33, 87], although there is no reference to *E. bieneusi* in the National Water Quality Management Strategy (NWQMS) of Australia or the Australian Drinking Water Guidelines (ADWG) contained within this strategy [53]. Given the lack of studies of *E. bieneusi* in Australia, it would seem prudent to gain insights into the epidemiology of this parasite in wildlife, water and the environment in Australia, to work toward an informed position on the public health significance of microsporidiosis associated with *E. bieneusi*. It would also be relevant to explore whether *E. bieneusi* survives currently used water treatment protocols [53].

## Conclusions

This study records for the first time *E. bieneusi* in sambar deer in Australia. The genotypes of *E. bieneusi* (D, J and Type IV) identified here have been detected previously in humans and water samples in other countries, which suggests that this deer species might act as a reservoir for genotypes that are transmissible to humans. As nothing is known about the occurrence, prevalence or distribution of *E. bieneusi* in other animals in Australia or its significance as a pathogen, future studies should elucidate the epidemiology of *E. bieneusi* in wildlife, water and the environment, in order to provide an informed position on its public health importance in this country. In the first instance, it would be important to focus on investigating whether other animals that are abundant in Melbourne’s water catchments carry *E. bieneusi* genotypes reported in the literature to infect humans. Other studies could be conducted to establish whether some of the genotypes recognised to be potentially zoonotic actually occur in humans in Australia.

## Additional files

Additional file 1: Table S1. *Enterocytozoon bieneusi* genotypes recorded from different animal species and water samples from published literature, representing nine distinct groups and two outgroups. The genotypes of *E. bieneusi* identified in this study are listed at the end of this table. (DOCX 74 kb)

Additional file 2: Table S2. Genotypes of *Enterocytozoon bieneusi* recorded previously in five species of deer worldwide and identified in sambar deer in the present study. (DOCX 69 kb)

Additional file 3: Table S3. Genotypes D, J and Type IV of *Enterocytozoon bieneusi* recorded in different animal species and water samples in previous studies. These genotypes were also recorded in the present study. (DOCX 359 kb)

## Abbreviations

AIC: Akaike information criteria
BI: Bayesian inference
ITS: Internal transcribed spacer of nuclear ribosomal DNA
*LSU*: Large subunit of nuclear ribosomal RNA gene
MCMC: Monte Carlo Markov Chain
pp: Posterior probability
*SSU*: Small subunit of nuclear ribosomal RNA gene
