# Supplementary material for: First detection and genetic characterisation of Enterocytozoon bieneusi in wild deer in Melbourne’s water catchments in Australia
Source: Parasit Vectors. 2018 Jan 3;11:2. doi: 10.1186/s13071-017-2577-7 (PMC5751821; doi:10.1186/s13071-017-2577-7)
Supplement: Supplementary file 1 — Enterocytozoon bieneusi genotypes recorded from different animal species and water samples from published literature, representing nine distinct groups and two outgroups. The genotypes of E. bieneusi identified in this study are listed at the end of this table. (DOCX 74 kb) [file 13071_2017_2577_MOESM1_ESM.docx]

**Additional file 1: Table S1** *Enterocytozoon bieneusi* genotypes recorded from different animal species and water samples from published literatures, representing nine distinct groups and two outgroups. The genotypes of *Enterocytozoon bieneusi* identified in this study were listed in the end of this table.

| Genotype name | GenBank accession no. | Origin | Latin name | Country | Group | | Reference |
| --- | --- | --- | --- | --- | --- | --- | --- |
| D | AF101200 | Human | *Homo sapiens* | Nigeria | Group 1a | | [1] |
| CHN3 | HM992511 | Cattle | *Bos taurus* | China | Group 1b | | [2] |
| isolate: UG2145 | AF502396 | Human | *H. sapiens* | Uganda | Group 1b | | [3] |
| NCF2 | KT750162 | Arctic fox | *V. lagopus* | China | Group 1b | | [4] |
| NCF3 | KT750163 | Arctic fox | *Vulpes lagopus* | China | Group 1b | | [4] |
| Peru6 | AY371281 | Human | *H. sapiens* | Peru | Group 1b | | [5] |
| Peru7 | AY371282 | Human | *H. sapiens* | Peru | Group 1b | | [5] |
| PtEb II | DQ425108 | Human | *H. sapiens* | Portugal | Group 1b | | [6] |
| PtEb V | DQ885581 | Kudo | *Tragelaphus strepsiceros* | Portugal | Group 1b | | [6] |
| PtEb VII | DQ885583 | Dog | *Canis familiaris* | Portugal | Group 1b | | [6] |
| R | AY945808 | Human | *H. sapiens* | Thailand | Group 1b | | [7] |
| S | AY945809 | Human | *H. sapiens* | Thailand | Group 1b | | [7] |
| S4 | FJ439679 | Human | *H. sapiens* | Malawi | Group 1b | | [8] |
| S5 | FJ439681 | Human | *H. sapiens* | Malawi | Group 1b | | [8] |
| SHW2 | KX190063 | Water | NA | China | Group 1b | | [9] |
| Wildboar2 | KF383397 | Wild boar | *Sus scrofa* | Poland | Group 1b | | [10] |
| Type IV | AF242478 | Human | *H. sapiens* | France | Group 1c | | [11] |
| E | AF135832 | Human | *H. sapiens* | Vietnam | Group 1d | | [12] |
| O | AF267145 | Human | *H. sapiens* | Thailand | Group 1e | | [7] |
| Q | AF267147 | Human | *H. sapiens* | Germany | Group 1f | | [13] |
| CAF1 | DQ683746 | Human | *H. sapiens* | Gabon | Group 1g | | [14] |
| Peru16 | EF014427 | Human | *H. sapiens* | Peru | Group 1h | | [15] |
| BEB6 | EU153584 | Red deer | *Cervus elaphus* | China | Group 2 | | [16] |
| J | AF135837 | Human | *H. sapiens* | China | Group 2 | | [2] |
| PtEb VIII | DQ885584 | Cat | *Felis catus* | Portugal | Group 3 | | [6] |
| S7 | FJ439683 | Human | *H. sapiens* | Netherlands | | Group 3 | [8] |
| WL4 | AY237212 | White-tailed deer | *Odocoileus virginianus* | USA | Group 3 | | [17] |
| WL1 | AY237209 | Raccoon | *P. lotor* | USA | Group 4 | | [18] |
| WL2 | AY237210 | Raccoon | *Procyon lotor* | USA | Group 4 | | [18] |
| CAF4 | DQ683757 | Human | *H. sapiens* | Cameroon | Group 5 | | [14] |
| KIN-3 | JQ437575 | Human | *H. sapiens* | Congo | Group 5 | | [19] |
| PtEb XII | DQ885588 | White-headed Marmoset | *Callithrix geoffroyi* | Portugal | Group 5 | | [6] |
| WW6 | JQ863274 | Waste water | NA | China | Group 6 | | [20] |
| WW7 | JQ863275 | Waste water | NA | China | Group 6 | | [20] |
| Nig4 | JN997480 | Human | *H. sapiens* | Nigeria | Group 7 | | [1] |
| Nig7 | JX524494 | Human | *H. sapiens* | Nigeria | Group 7 | | [1] |
| Horse 2 | GQ406054 | Horse | *Equus ferus caballus* | Colombia | Group 8 | | [21] |
| KB-5 | JF681179 | Olive baboon | *Papio anubis* | Kenya | Group 8 | | [22] |
| CD5 | KJ668732 | Dog | *C. familiaris* | China | Group 9 | | [23] |
| CM4 | KF543866 | Goat | *Capra aegagrus hircus* | China | Group 9 | | [24] |
| CD8 | KJ668735 | Dog | *C. familiaris* | China | Outgroup | | [23] |
| PtEb IX | DQ885585 | Dog | *C. familiaris* | USA | Outgroup | | [25] |
| D | MF693831 | Sambar deer | *Rusa unicolor* | AUS |  | | This study |
| J | MF693833 | Sambar deer | *R. unicolor* | AUS |  | | This study |
| MWC_d1 | MF496204 | Sambar deer | *R. unicolor* | AUS |  | | This study |
| MWC_d2 | MF496203 | Sambar deer | *R. unicolor* | AUS |  | | This study |
| Type IV | MF693832 | Sambar deer | *R. unicolor* | AUS |  | | This study |

GenBank No. AF502396 showed no genotype name, thus isolate number was used. NA= not available.

**References**

1. Akinbo FO, Okaka CE, Omoregie R, Adamu H, Xiao L. Unusual *Enterocytozoon bieneusi* genotypes and *Cryptosporidium* hominis subtypes in HIV-infected patients on highly active antiretroviral therapy. Am J Trop Med Hyg. 2013;89(1):157-61.

2. Zhang X, Wang Z, Su Y, Liang X, Sun X, Peng S, et al. Identification and genotyping of *Enterocytozoon bieneusi* in China. J Clin Microbiol. 2011;49(5):2006-8.

3. Tumwine JK, Kekitiinwa A, Nabukeera N, Akiyoshi DE, Buckholt MA, Tzipori S. *Enterocytozoon bieneusi* among children with diarrhea attending Mulago Hospital in Uganda. Am J Trop Med Hyg. 2002;67(3):299-303.

4. Zhang XX, Cong W, Lou ZL, Ma JG, Zheng WB, Yao QX, et al. Prevalence, risk factors and multilocus genotyping of *Enterocytozoon bieneusi* in farmed foxes (*Vulpes lagopus*), northern China. Parasit Vectors. 2016;9(1):72.

5. Sulaiman IM, Bern C, Gilman R, Cama V, Kawai V, Vargas D, et al. A molecular biologic study of *Enterocytozoon bieneusi* in HIV-infected patients in Lima, Peru. J Eukaryot Microbiol. 2003;50 Suppl:591-6.

6. Lobo ML, Xiao L, Antunes F, Matos O. Microsporidia as emerging pathogens and the implication for public health: a 10-year study on HIV-positive and -negative patients. Int J Parasitol. 2012;42(2):197-205.

7. Leelayoova S, Subrungruang I, Suputtamongkol Y, Worapong J, Petmitr PC, Mungthin M. Identification of genotypes of *Enterocytozoon bieneusi* from stool samples from human immunodeficiency virus-infected patients in Thailand. J Clin Microbiol. 2006;44(8):3001-4.

8. ten Hove RJ, Van Lieshout L, Beadsworth MB, Perez MA, Spee K, Claas EC, et al. Characterization of genotypes of *Enterocytozoon bieneusi* in immunosuppressed and immunocompetent patient groups. J Eukaryot Microbiol. 2009;56(4):388-93.

9. Huang C, Hu Y, Wang L, Wang Y, Li N, Guo Y, et al. Environmental transport of emerging human-pathogenic *Cryptosporidium* species and subtypes through combined sewer overflow and wastewater. Appl Environ Microbiol. 2017;AEM-00682.

10. Němejc K, Sak B, Květoňová D, Hanzal V, Janiszewski P, Forejtek P, et al. Prevalence and diversity of *Encephalitozoon* spp. and *Enterocytozoon bieneusi* in wild boars (*Sus scrofa*) in Central Europe. Parasitol Res. 2014;113(2):761-7.

11. Liguory O, Sarfati C, Derouin F, Molina JM. Evidence of different *Enterocytozoon bieneusi* genotypes in patients with and without human immunodeficiency virus infection. J Clin Microbiol. 2001;39(7):2672-4.

12. Espern A, Morio F, Miegeville M, Illa H, Abdoulaye M, Meyssonnier V, et al. Molecular study of microsporidiosis due to *Enterocytozoon bieneusi* and *Encephalitozoon intestinalis* among human immunodeficiency virus-infected patients from two geographical areas: Niamey, Niger, and Hanoi, Vietnam. J Clin Microbiol. 2007;45(9):2999-3002.

13. Dengjel B, Zahler M, Hermanns W, Heinritzi K, Spillmann T, Thomschke A, et al. Zoonotic potential of *Enterocytozoon bieneusi*. J Clin Microbiol. 2001;39(12):4495-9.

14. Breton J, Bart-Delabesse E, Biligui S, Carbone A, Seiller X, Okome-Nkoumou M, et al. New highly divergent rRNA sequence among biodiverse genotypes of *Enterocytozoon bieneusi* strains isolated from humans in Gabon and Cameroon. J Clin Microbiol. 2007;45(8):2580-9.

15. Cama VA, Pearson J, Cabrera L, Pacheco L, Gilman R, Meyer S, et al. Transmission of *Enterocytozoon bieneusi* between a child and guinea pigs. J Clin Microbiol. 2007;45(8):2708-10.

16. Zhao W, Zhang W, Wang R, Liu W, Liu A, Yang D, et al. *Enterocytozoon bieneusi* in sika deer (*Cervus nippon*) and red deer (*Cervus elaphus*): deer specificity and zoonotic potential of ITS genotypes. Parasitol Res. 2014;113(11):4243-50.

17. Guo Y, Alderisio KA, Yang W, Cama V, Feng Y, Xiao L. Host specificity and source of *Enterocytozoon bieneusi* genotypes in a drinking source watershed. Appl Environ Microbiol. 2014;80(1):218-25.

18. Sulaiman IM, Fayer R, Yang C, Santín M, Matos O, Xiao L. Molecular characterization of *Enterocytozoon bieneusi* in cattle indicates that only some isolates have zoonotic potential. Parasitol Res. 2004;92(4):328-34.

19. Wumba R, Longo-Mbenza B, Menotti J, Mandina M, Kintoki F, Situakibanza NH, et al. Epidemiology, clinical, immune, and molecular profiles of microsporidiosis and cryptosporidiosis among HIV/AIDS patients. Int J Gen Med. 2012;5:603-11.

20. Li N, Xiao L, Wang L, Zhao S, Zhao X, Duan L, et al. Molecular surveillance of *Cryptosporidium* spp., *Giardia duodenalis*, and *Enterocytozoon bieneusi* by genotyping and subtyping parasites in wastewater. PLoS Negl Trop Dis. 2012;6(9):e1809.

21. Santín M, Vecino JAC, Fayer R. A zoonotic genotype of *Enterocytozoon bieneusi* in horses. J Parasitol. 2010;96(1):157-61.

22. Li W, Kiulia NM, Mwenda JM, Nyachieo A, Taylor MB, Zhang X, et al. *Cyclospora papionis*, *Cryptosporidium hominis,* and human-pathogenic *Enterocytozoon bieneusi* in captive baboons in Kenya. J Clin Microbiol. 2011;49(12):4326-9.

23. Karim MR, Dong H, Yu F, Jian F, Zhang L, Wang R, et al. Genetic diversity in *Enterocytozoon bieneusi* isolates from dogs and cats in China: host specificity and public health implications. J Clin Microbiol. 2014;52(9):3297-302.

24. Shi K, Li M, Wang X, Li J, Karim MR, Wang R, et al. Molecular survey of *Enterocytozoon bieneusi* in sheep and goats in China. Parasit Vectors. 2016;9(1):23.

25. Feng Y, Li N, Dearen T, Lobo ML, Matos O, Cama V, et al. Development of a multilocus sequence typing tool for high-resolution genotyping of *Enterocytozoon bieneusi*. Appl Environ Microbiol. 2011;77(14):4822-8.
