# Supplementary material for: First detection and genetic characterisation of Enterocytozoon bieneusi in wild deer in Melbourne’s water catchments in Australia
Source: Parasit Vectors. 2018 Jan 3;11:2. doi: 10.1186/s13071-017-2577-7 (PMC5751821; doi:10.1186/s13071-017-2577-7)
Supplement: Supplementary file 2 — Genotypes of Enterocytozoon bieneusi recorded previously in five species of deer worldwide and identified in sambar deer in the present study. (DOCX 69 kb) [file 13071_2017_2577_MOESM2_ESM.docx]

**Additional file 2: Table S2**. Genotypes of *Enterocytozoon bieneusi* recorded previously in five species of deer worldwide and identified in Sambar deer in the present study.

| Genotype name (synonym) | GenBank accession no. | Deer species | Scientific name | Country | Reference |
| --- | --- | --- | --- | --- | --- |
| BEB6 | KU852483 | Hog deer | *Axis porcinus* | China | [1] |
| CHS9 | KU852481 | Hog deer | *A. porcinus* | China | [1] |
| BEB6 | KP057601 | Père David’s deer | *Elaphurus davidianus* | China | [2] |
| COS-I | KP057602 | Père David’s deer | *E. davidianus* | China | [2] |
| COS-II | KP057603 | Père David’s deer | *E. davidianus* | China | [2] |
| EbpA | KP057600 | Père David’s deer | *E. davidianus* | China | [2] |
| EbpC | KP057599 | Père David’s deer | *E. davidianus* | China | [2] |
| Type IV | KP057598 | Père David’s deer | *E. davidianus* | China | [2] |
| BEB6 | KX383616 | Red deer | *Cervus elaphus* | China | [3] |
| BEB6 | KU852482 | Red deer | *C. elaphus* | China | [1] |
| BEB6 | EU153584 | Red deer | *C. elaphus* | China | [4] |
| HLJD-I | KJ556984 | Red deer | *C. elaphus* | China | [4] |
| HLJD-II | KJ556985 | Red deer | *C. elaphus* | China | [4] |
| HLJD-III | KJ556986 | Red deer | *C. elaphus* | China | [4] |
| HLJD-IV | KJ556987 | Red deer | *C. elaphus* | China | [4] |
| HLJD-V | KJ556988 | Red deer | *C. elaphus* | China | [4] |
| JLD-IV | KX383632 | Red deer | *C. elaphus* | China | [3] |
| JLD-XIII | KX383645 | Red deer | *C. elaphus* | China | [3] |
| BEB6 | KX383614 | Sika deer | *Cervus nippon* | China | [3] |
| BEB6 | KX383615 | Sika deer | *C. nippon* | China | [3] |
| BEB6 | KU852485 | Sika deer | *C. nippon* | China | [1] |
| BEB6 | KR815519 | Sika deer | *C. nippon* | China | [5] |
| BEB6 | EU153584 | Sika deer | *C. nippon* | China | [4] |
| CHN-DC1 | KR815516 | Sika deer | *C. nippon* | China | [5] |
| COS-I | KX383622 | Sika deer | *C. nippon* | China | [3] |
| CS03 | KU852479 | Sika deer | *C. nippon* | China | [1] |
| D | KX383624 | Sika deer | *C. nippon* | China | [3] |
| EbpA | KX383623 | Sika deer | *C. nippon* | China | [3] |
| EbpC | KX383619 | Sika deer | *C. nippon* | China | [3] |
| EbpC | KX383620 | Sika deer | *C. nippon* | China | [3] |
| EbpC | KR815517 | Sika deer | *C. nippon* | China | [5] |
| HLJD-I | KX383617 | Sika deer | *C. nippon* | China | [3] |
| HLJD-I | KX383618 | Sika deer | *C. nippon* | China | [3] |
| HLJD-I | KJ556984 | Sika deer | *C. nippon* | China | [4] |
| HLJD-II | KJ556985 | Sika deer | *C. nippon* | China | [4] |
| HLJD-III | KJ556986 | Sika deer | *C. nippon* | China | [4] |
| HLJD-IV | KX383621 | Sika deer | *C. nippon* | China | [3] |
| HLJD-IV | KJ556987 | Sika deer | *C. nippon* | China | [4] |
| HLJD-V | KJ556988 | Sika deer | *C. nippon* | China | [4] |
| HND-I | KX383628 | Sika deer | *C. nippon* | China | [3] |
| HND-II | KX383630 | Sika deer | *C. nippon* | China | [3] |
| HND-III | KX383636 | Sika deer | *C. nippon* | China | [3] |
| HND-IV | KX383641 | Sika deer | *C. nippon* | China | [3] |
| J | KR815512 | Sika deer | *C. nippon* | China | [5] |
| JLD-1 | KR815518 | Sika deer | *C. nippon* | China | [5] |
| JLD-2 | KR815515 | Sika deer | *C. nippon* | China | [5] |
| JLD-3 | KR815513 | Sika deer | *C. nippon* | China | [5] |
| JLD-I | KX383625 | Sika deer | *C. nippon* | China | [3] |
| JLD-II | KX383626 | Sika deer | *C. nippon* | China | [3] |
| JLD-II | KX383627 | Sika deer | *C. nippon* | China | [3] |
| JLD-III | KX383629 | Sika deer | *C. nippon* | China | [3] |
| JLD-IV | KX383631 | Sika deer | *C. nippon* | China | [3] |
| JLD-IX | KX383639 | Sika deer | *C. nippon* | China | [3] |
| JLD-V | KX383633 | Sika deer | *C. nippon* | China | [3] |
| JLD-VI | KX383634 | Sika deer | *C. nippon* | China | [3] |
| JLD-VI | KX383635 | Sika deer | *C. nippon* | China | [3] |
| JLD-VII | KX383637 | Sika deer | *C. nippon* | China | [3] |
| JLD-VIII | KX383638 | Sika deer | *C. nippon* | China | [3] |
| JLD-X | KX383640 | Sika deer | *C. nippon* | China | [3] |
| JLD-XI | KX383642 | Sika deer | *C. nippon* | China | [3] |
| JLD-XI | KX383643 | Sika deer | *C. nippon* | China | [3] |
| JLD-XII | KX383644 | Sika deer | *C. nippon* | China | [3] |
| JLD-XIV | KX383646 | Sika deer | *C. nippon* | China | [3] |
| JLD-XIV | KX383647 | Sika deer | *C. nippon* | China | [3] |
| KIN-1 | KR815514 | Sika deer | *C. nippon* | China | [5] |
| DeerEb1 | KJ867480 | White-tailed deer | *Odocoileus virginianus* | USA | [6] |
| DeerEb2 | KJ867481 | White-tailed deer | *O. virginianus* | USA | [6] |
| DeerEb3 | KJ867482 | White-tailed deer | *O. virginianus* | USA | [6] |
| DeerEb4 | KJ867483 | White-tailed deer | *O. virginianus* | USA | [6] |
| DeerEb5 | KJ867484 | White-tailed deer | *O. virginianus* | USA | [6] |
| DeerEb6 | KJ867485 | White-tailed deer | *O. virginianus* | USA | [6] |
| DeerEb7 | KJ867486 | White-tailed deer | *O. virginianus* | USA | [6] |
| DeerEb8 | KJ867487 | White-tailed deer | *O. virginianus* | USA | [6] |
| DeerEb9 | KJ867488 | White-tailed deer | *O. virginianus* | USA | [6] |
| DeerEb10 | KJ867489 | White-tailed deer | *O. virginianus* | USA | [6] |
| DeerEb11 | KJ867490 | White-tailed deer | *O. virginianus* | USA | [6] |
| DeerEb12 | KJ867491 | White-tailed deer | *O. virginianus* | USA | [6] |
| DeerEb13 | KJ867492 | White-tailed deer | *O. virginianus* | USA | [6] |
| I | KJ867479 | White-tailed deer | *O. virginianus* | USA | [6] |
| J | KJ867478 | White-tailed deer | *O. virginianus* | USA | [6] |
| LW1 | KJ867477 | White-tailed deer | *O. virginianus* | USA | [6] |
| WL4 | AY237212 | White-tailed deer | *O. virginianus* | USA | [7] |
| WL4 | KJ867476 | White-tailed deer | *O. virginianus* | USA | [6] |
| WL18 | KF591680 | White-tailed deer | *O. virginianus* | USA | [7] |
| WL19 | KF591681 | White-tailed deer | *O. virginianus* | USA | [7] |
| D | MF693831 | Sambar deer | *Rusa unicolor* | AUS | This study |
| J | MF693833 | Sambar deer | *R. unicolor* | AUS | This study |
| MWC_d1 | MF496204 | Sambar deer | *R. unicolor* | AUS | This study |
| MWC_d2 | MF496203 | Sambar deer | *R. unicolor* | AUS | This study |
| Type IV | MF693832 | Sambar deer | *R. unicolor* | AUS | This study |

**References**

1. Li W, Deng L, Yu X, Zhong Z, Wang Q, Liu X, et al. Multilocus genotypes and broad host-range of *Enterocytozoon bieneusi* in captive wildlife at zoological gardens in China. Parasit Vectors. 2016;9(1):395.

2. Zhang Z, Huang J, Karim MR, Zhao J, Dong H, Ai W, et al. Zoonotic *Enterocytozoon bieneusi* genotypes in Père David's deer (*Elaphurus davidianus*) in Henan, China. Exp Parasitol. 2015;155:46-8.

3. Huang J, Zhang Z, Yang Y, Wang R, Zhao J, Jian F, et al. New genotypes of *Enterocytozoon bieneusi* isolated from sika deer and red deer in China. Front Microbiol. 2017;8:879.

4. Zhao W, Zhang W, Wang R, Liu W, Liu A, Yang D, et al. *Enterocytozoon bieneusi* in sika deer (*Cervus nippon*) and red deer (*Cervus elaphus*): deer specificity and zoonotic potential of ITS genotypes. Parasitol Res. 2014;113(11):4243-50.

5. Zhang XX, Cong W, Liu GH, Ni XT, Ma JG, Zheng WB, et al. Prevalence and genotypes of *Enterocytozoon bieneusi* in sika deer in Jilin province, northeastern China. Acta Parasitol. 2016;61(2):382-8.

6. Santín M, Fayer R. *Enterocytozoon bieneusi, Giardia,* and *Cryptosporidium* infecting white-tailed deer. J Eukaryot Microbiol. 2015;62(1):34-43.

7. Guo Y, Alderisio KA, Yang W, Cama V, Feng Y, Xiao L. Host specificity and source of *Enterocytozoon bieneusi* genotypes in a drinking source watershed. Appl Environ Microbiol. 2014;80(1):218-25.
