# Supplementary material for: First detection and genetic characterisation of Enterocytozoon bieneusi in wild deer in Melbourne’s water catchments in Australia
Source: Parasit Vectors. 2018 Jan 3;11:2. doi: 10.1186/s13071-017-2577-7 (PMC5751821; doi:10.1186/s13071-017-2577-7)
Supplement: Supplementary file 3 — Genotypes D, J and Type IV of Enterocytozoon bieneusi recorded in different animal species and water samples in previous studies. These genotypes were also recorded in the present study. (DOCX 359 kb) [file 13071_2017_2577_MOESM3_ESM.docx]

**Additional file 3:** **Table S3.** Genotypes D, J and Type IV of *Enterocytozoon bieneusi* recorded in different animal species and water samples in previous studies. These genotypes were also recorded in the present study.

| Genotype name (synonym) | GenBank accession no. | Host species | Latin name | Country | Reference |
| --- | --- | --- | --- | --- | --- |
| D | KT943972 | Chicken | *Gallus gallus domesticus* | Brazil | [1] |
| D | KT943972 | Chicken | *G. g. domesticus* | Brazil | [1] |
| D | KT943972 | Chicken | *G. g. domesticus* | Brazil | [2] |
| D | KT943972 | Chicken | *G. g. domesticus* | Brazil | [2] |
| D | AF101200 | Common crane | *Grus grus* | China | [3] |
| D | AF101200 | Common quail | *Coturnix coturnix* | Brazil | [1] |
| D | AF101200 | Common quail | *C. coturnix* | Brazil | [2] |
| D | DQ793213 | Gyr-Peregrine falcon | *Falco rusticolus x Falco cherrug* | Abu Dhabi | [4] |
| D | DQ793213 | Gyr-Saker falcon | *F. rusticolus x Falco peregrinus* | Abu Dhabi | [4] |
| D | DQ793213 | Gyrfalcon | *F. rusticolus* | Abu Dhabi | [4] |
| D | DQ793213 | Lanner falcon | *Falco biarmicus* | Abu Dhabi | [4] |
| D | DQ793213 | Peregrine falcon | *F. peregrinus* | Abu Dhabi | [4] |
| D | JF776168 | Pigeon | *Columba livia* | Iran | [5] |
| D | AF101200 | Red-crowned crane | *Grus japonensis* | China | [3] |
| D | KT267295 | Red-headed lovebird | *Agapornis pullarius* | China | [6] |
| D | KX375799 | Rook | *Corvus frugilegus* | Poland | [7] |
| D | KY012355 | Swan goose | *Anser cygnoides* | Brazil | [2] |
| D | KY012355 | Swan goose | *A. cygnoides* | Brazil | [8] |
| D | KT750166 | Arctic fox | *Vulpes lagopus* | China | [9] |
| D | AF101200 | Arctic fox | *V. lagopus* | China | [10] |
| D | KR062135 | Bank vole | *Myodes glareolus* | Poland | [11] |
| D | AF101200 | Bornean orangutan | *P. pygmaeus* | China | [12] |
| D | KJ728787 | Bornean orangutan | *P. pygmaeus* | China | [13] |
| D | AF101200 | Bornean orangutan | *Pongo pygmaeus* | Indonesia | [14] |
| D | KJ668727 | Cat | *Felis catus* | China | [15] |
| D | AF101200 | Cat | *F. catus* | China | [16] |
| D | KF305583 | Cat | *F. catus* | China | [17] |
| D | AF101200 | Cat | *F. catus* | Thailand | [18] |
| D | KF675191 | Cattle | *B. taurus* | Argentina | [19] |
| D | KT984496 | Cattle | *Bos taurus* | Brazil | [20] |
| D | AF101200 | Cattle | *B. taurus* | China | [21] |
| D | AF101200 | Cattle | *B. taurus* | China | [22] |
| D | KU531574 | Cattle | *B. taurus* | China | [23] |
| D | AF101200 | Cattle | *B. taurus* | Korea | [24] |
| D (CEbC) | EF139197 | Cattle | *B. taurus* | Korea | [24] |
| D | AF101200 | Cattle | *B. taurus* | Korea | [25] |
| D | JQ923453 | Cattle | *B. taurus* | South Africa | [26] |
| D | DQ683755 | Crab-eating macaque | *Macaca fascicularis* | China | [27] |
| D | AF101200 | Cynomolgus monkey | *M. fascicularis* | China | [12] |
| D | KF305583 | Cynomolgus monkey | *M. fascicularis* | China | [28] |
| D | KJ668727 | Dog | *Canis familiaris* | China | [15] |
| D | AF101200 | Dog | *C. familiaris* | China | [16] |
| D | KF305583 | Dog | *C. familiaris* | China | [17] |
| D (PtEb VI) | DQ885582 | Dog | *C. familiaris* | Portugal | [29] |
| D | KT325924 | Domestic rabbit | *Oryctolagus cuniculus* | China | [30] |
| D | KU557672 | Donkey | *Equus africanus asinus* | China | [31] |
| D | AF101200 | East-European house mouse | *Mus musculus musculus* | Czech Republic / Germany | [32] |
| D | KX670582 | Eurasian wild boar | *Sus scrofa* | China | [33] |
| D | AF101200 | Goat | *Capra aegagrus hircus* | China | [34] |
| D | KP262379 | Goat | *C. a. hircus* | China | [35] |
| D | KJ728787 | Golden monkey | *Cercopithecus kandti* | China | [13] |
| D | AF101200 | Golden snub-nosed monkey | *R. roxellana* | China | [12] |
| D | KM591956 | Golden snub-nosed monkey | *R. roxellana* | China | [36] |
| D | KM591955 | Golden snub-nosed monkey | *Rhinopithecus roxellana* | China | [36] |
| D | KU604932 | Golden snub-nosed monkey | *R. roxellana* | China | [37] |
| D | KU852470 | Golden snub-nosed monkey | *R. roxellana* | China | [38] |
| D | AF101200 | Golden takin | *Budorcas taxicolor bedfordi* | China | [39] |
| D | JQ837799 | Western lowland gorilla | *G. g. gorilla* | Central African Republic | [40] |
| D | JQ837800 | Western lowland gorilla | *G. g. gorilla* | Central African Republic | [40] |
| D | JQ837798 | Western lowland gorilla | *Gorilla gorilla gorilla* | Central African Republic | [40] |
| D | AF101200 | Green monkey | *C. sabaeus* | China | [12] |
| D | KJ728787 | Green monkey | *Chlorocebus sabaeus* | China | [13] |
| D | AF101200 | Hamadryas baboon | *P. hamadryas* | China | [12] |
| D | KJ728787 | Hamadryas baboon | *Papio hamadryas* | China | [13] |
| D | KM591953 | Hamadryas baboon | *P. hamadryas* | China | [36] |
| D | KM591954 | Hamadryas baboon | *P. hamadryas* | China | [36] |
| D | KT267281 | Hippopotamus | *Hippopotamus amphibius* | China | [6] |
| D | KU194595 | Horse | *Equus ferus caballus* | China | [41] |
| D | KX276705 | Horse | *E. f. caballus* | China | [42] |
| D | GQ406055 | Horse | *E. f. caballus* | Colombia | [43] |
| D | AF101200 | Horse | *E. f. caballus* | Czech Republic | [44] |
| D | JF927954 | Human | *Homo sapiens* | Brazil | [45] |
| D | AF101200 | Human | *H. sapiens* | Cameroon | [46] |
| D | JQ029731 | Human | *H. sapiens* | China | [47] |
| D | JX994272 | Human | *H. sapiens* | China | [48] |
| D | JQ029731 | Human | *H. sapiens* | China | [49] |
| D | AF101200 | Human | *H. sapiens* | Czech Republic | [50] |
| D | AF101200 | Human | *H. sapiens* | England | [51] |
| D | AF101200 | Human | *H. sapiens* | Gabon | [46] |
| D | JQ923453 | Human | *H. sapiens* | India | [52] |
| D | AF101200 | Human | *H. sapiens* | Iran | [53] |
| D | KJ700425 | Human | *H. sapiens* | Iran | [54] |
| D | KJ700427 | Human | *H. sapiens* | Iran | [54] |
| D | KJ700429 | Human | *H. sapiens* | Iran | [54] |
| D | KJ700430 | Human | *H. sapiens* | Iran | [54] |
| D | KJ700431 | Human | *H. sapiens* | Iran | [54] |
| D | KJ700434 | Human | *H. sapiens* | Iran | [54] |
| D | KJ700435 | Human | *H. sapiens* | Iran | [54] |
| D | KJ700436 | Human | *H. sapiens* | Iran | [54] |
| D | KJ700437 | Human | *H. sapiens* | Iran | [54] |
| D | AF101200 | Human | *H. sapiens* | Iran | [55] |
| D | KY859474 | Human | *H. sapiens* | Iran | [56] |
| D | KY859475 | Human | *H. sapiens* | Iran | [56] |
| D | KY859476 | Human | *H. sapiens* | Iran | [56] |
| D | KY859477 | Human | *H. sapiens* | Iran | [56] |
| D | KY859478 | Human | *H. sapiens* | Iran | [56] |
| D | KY859479 | Human | *H. sapiens* | Iran | [56] |
| D | KY859480 | Human | *H. sapiens* | Iran | [56] |
| D | KY859481 | Human | *H. sapiens* | Iran | [56] |
| D | KY859482 | Human | *H. sapiens* | Iran | [56] |
| D | KY859483 | Human | *H. sapiens* | Iran | [56] |
| D | KY859484 | Human | *H. sapiens* | Iran | [56] |
| D | KY859485 | Human | *H. sapiens* | Iran | [56] |
| D | KY859486 | Human | *H. sapiens* | Iran | [56] |
| D | KY859487 | Human | *H. sapiens* | Iran | [56] |
| D | KY859488 | Human | *H. sapiens* | Iran | [56] |
| D | KY859489 | Human | *H. sapiens* | Iran | [56] |
| D | KY859490 | Human | *H. sapiens* | Iran | [56] |
| D | KY859491 | Human | *H. sapiens* | Iran | [56] |
| D | KY859492 | Human | *H. sapiens* | Iran | [56] |
| D | AF101200 | Human | *H. sapiens* | Malawi | [57] |
| D | AF101200 | Human | *H. sapiens* | Netherlands | [57] |
| D | AF101200 | Human | *H. sapiens* | Nigeria | [58] |
| D | AF101200 | Human | *H. sapiens* | Nigeria | [59] |
| D | JQ923453 | Human | *H. sapiens* | Nigeria | [60] |
| D | JX683802 | Human | *H. sapiens* | Nigeria | [61] |
| D (Peru 9) | AY371284 | Human | *H. sapiens* | Peru | [62] |
| D (Peru 9) | AY371284 | Human | *H. sapiens* | Peru | [63] |
| D (Peru 9) | AY371284 | Human | *H. sapiens* | Peru | [64] |
| D | AF101200 | Human | *H. sapiens* | Poland | [65] |
| D | AF101200 | Human | *H. sapiens* | Portugal | [66] |
| D | AF101200 | Human | *H. sapiens* | Russia | [67] |
| D (Peru 9) | AY371284 | Human | *H. sapiens* | Russia | [67] |
| D (WL 8) | AY237216 | Human | *H. sapiens* | Russia | [67] |
| D | AF101200 | Human | *H. sapiens* | Thailand | [68] |
| D | AF101200 | Human | *H. sapiens* | Thailand | [69] |
| D | AF101200 | Human | *H. sapiens* | Thailand | [70] |
| D | AF101200 | Human | *H. sapiens* | Thailand | [71] |
| D | AF101200 | Human | *H. sapiens* | Tunisia | [72] |
| D | AF101200 | Human | *H. sapiens* | Vietnam | [73] |
| D | AF101200 | Hussar monkey | *Erythrocebus patas* | China | [12] |
| D | KJ728787 | Hussar monkey | *E. patas* | China | [13] |
| D | AF101200 | King colobus | *Colobus polykomos* | China | [12] |
| D | KJ728787 | King colobus | *C. polykomos* | China | [13] |
| D | AF101200 | Leaf monkey | *Presbytis leucocephalus* | China | [12] |
| D | KT267298 | Lion | *Pamthera leo* | China | [6] |
| D | KU852469 | Lion | *P. leo* | China | [38] |
| D | AF101200 | Long-tailed chinchilla | *Chinchilla lanigera* | China | [41] |
| D (WL 8) | AY237216 | Muskrat | *Ondatra zibethicus* | USA | [74] |
| D (WL 8) | AY237216 | North American beaver | *Castor canadensis* | USA | [74] |
| D | AF101200 | North American river otter | *Lontra canadensis* | USA | [75] |
| D | AF101200 | Northern white-cheeked gibbon | *Nomascus leucogenys* | China | [12] |
| D | KJ728787 | Northern white-cheeked gibbon | *N. leucogenys* | China | [13] |
| D | KX905211 | Northern white-cheeked gibbon | *N. leucogenys* | China | [76] |
| D | KX423961 | Northern white-cheeked gibbon | *N. leucogenys* | China | [38] |
| D | KJ728787 | Olive baboon | *Papio anubis* | China | [13] |
| D | KU852471 | Olive baboon | *P. anubis* | China | [38] |
| D | DQ683751 | Olive baboon | *P. anubis* | Kenya | [77] |
| D | AF101200 | Pig | *Sus scrofa domesticus* | China | [78] |
| D | JF776168 | Pig | *S. s. domesticus* | China | [79] |
| D (Peru 9) | AY371284 | Pig | *S. s. domesticus* | Czech Republic | [80] |
| D | AF101200 | Pig | *S. s. domesticus* | Czech Republic | [80] |
| D | AF101200 | Pig | *S. s. domesticus* | Japan | [81] |
| D | AF101200 | Pig | *S. s. domesticus* | Thailand | [71] |
| D | KU852472 | Raccoon | *Procyon lotor* | China | [38] |
| D (WL 8) | AY237216 | Raccoon | *P. lotor* | USA | [74] |
| D | AF101200 | Raccoon dog | *Nyctereutes procyonoides* | China | [10] |
| D | AF101200 | Raccoon dog | *N. procyonoides* | China | [82] |
| D | KU847361 | Raccoon dog | *N. procyonoides* | China | [83] |
| D | AF101200 | Red fox | *V. vulpes* | China | [82] |
| D (WL 8) | AY237216 | Red fox | *V. vulpes* | USA | [74] |
| D | AF101200 | Red-bellied tree squirrel | *Callosciurus erythraeus* | China | [84] |
| D | AB470282 | Rex rabbit | *O. cuniculus* | China | [85] |
| D | AF101200 | Rhesus macaque | *Macaca mulatta* | China | [12] |
| D | KJ728787 | Rhesus macaque | *M. mulatta* | China | [13] |
| D | KF305583 | Rhesus macaque | *M. mulatta* | China | [28] |
| D | KM591957 | Rhesus macaque | *M. mulatta* | China | [36] |
| D | KM591958 | Rhesus macaque | *M. mulatta* | China | [36] |
| D | KM591959 | Rhesus macaque | *M. mulatta* | China | [36] |
| D | KM591960 | Rhesus macaque | *M. mulatta* | China | [36] |
| D | KM591961 | Rhesus macaque | *M. mulatta* | China | [36] |
| D | KM591962 | Rhesus macaque | *M. mulatta* | China | [36] |
| D | KX905208 | Rhesus macaque | *M. mulatta* | China | [76] |
| D | KX905209 | Rhesus macaque | *M. mulatta* | China | [76] |
| D | KX905210 | Rhesus macaque | *M. mulatta* | China | [76] |
| D | AF101200 | Rhesus macaque | *M. mulatta* | England | [86] |
| D | MF693831 | Sambar deer | *Rusa unicolor* | Australia | This study |
| D | AF101200 | Sheep | *Ovis aries* | China | [34] |
| D | KT267294 | Siberian tiger | *Panthera tigris altaica* | China | [6] |
| D | KX383624 | Sika deer | *Cervus nippon* | China | [87] |
| D | KR062137 | Striped field mouse | *Apodemus agrarius* | Poland | [11] |
| D | KR062136 | Striped field mouse | *A. agrarius* | Poland | [11] |
| D | AF101200 | Sumatran orangutan | *Pongo abelii* | Indonesia | [14] |
| D | AF101200 | Swine | *S. s. domesticus* | USA | [88] |
| D (PigEBITS9) | AF348477 | Swine | *S. s. domesticus* | USA | [88] |
| D | AF101200 | West-European house mouse | *Mus musculus domesticus* | Czech Republic / Germany | [32] |
| D | KF305583 | White-headed langur | *Trachypithecus poliocephalus* | China | [28] |
| D | KF383389 | Wild boar | *S. scrofa* | Austria | [89] |
| D | KF383390 | Wild boar | *S. scrofa* | Czech Republic | [89] |
| D | KF383391 | Wild boar | *S. scrofa* | Czech Republic | [89] |
| D | KF383392 | Wild boar | *S. scrofa* | Czech Republic | [89] |
| D | KF383393 | Wild boar | *S. scrofa* | Czech Republic | [89] |
| D | KF383395 | Wild boar | *S. scrofa* | Czech Republic | [89] |
| D | KF383394 | Wild boar | *S. scrofa* | Slovak Republic | [89] |
| D | KR062139 | Yellow necked mouse | *Apodemus flavicollis* | Poland | [11] |
| D | KR062138 | Yellow necked mouse | *A. flavicollis* | Poland | [11] |
| D | AF101200 | Water | NA | China | [90] |
| D | AF101200 | Water | NA | China | [91] |
| D | AF101200 | Water | NA | China | [92] |
| D | AF023245 | Water | NA | Spain | [93] |
| D | AF101200 | Water | NA | China | [94] |
| J | AF135837 | Chicken | *G. g. domesticus* | Germany | [95] |
| J | JF776170 | Pigeon | *C. livia* | Iran | [5] |
| J | KT267279 | Alpaca | *Vicugna pacos* | China | [6] |
| J | KT267292 | Big ear sheep | unknown | China | [6] |
| J | AF135837 | Cattle | *B. taurus* | USA | [96] |
| J | AF135837 | Cattle | *B. taurus* | China | [97] |
| J | AF135837 | Cattle | *B. taurus* | USA | [98] |
| J (PtEb X) | DQ885586 | Cattle | *B. taurus* | Portugal | [29] |
| J | AF135837 | Cattle | *B. taurus* | China | [99] |
| J | AF135837 | Cattle | *B. taurus* | Algeria | [100] |
| J | AF135837 | Cattle | *B. taurus* | China | [101] |
| J | AF135837 | Cattle | *B. taurus* | China | [102] |
| J (CEbB) | EF139196 | Cattle | *B. taurus* | Korea | [24] |
| J | KF675193 | Cattle | *B. taurus* | Argentina | [19] |
| J (BEB1) | AY331005 | Cattle | *B. taurus* | USA | [74] |
| J | AF135837 | Cattle | *B. taurus* | China | [103] |
| J | AF135837 | Cattle | *B. taurus* | Germany | [104] |
| J | AF135837 | Cattle | *B. taurus* | China | [97] |
| J (BEB1) | AY331005 | Cattle | *B. taurus* | USA | [105] |
| J (BEB1) | AF135837 | Cattle | *B. taurus* | USA | [106] |
| J | AF135837 | Cattle | *B. taurus* | China | [21] |
| J | AF135837 | Cattle | *B. taurus* | China | [22] |
| J | KU531571 | Cattle | *B. taurus* | China | [23] |
| J | KM110054 | Cattle | *B. taurus* | China | [103] |
| J | AF135837 | Cattle | *B. taurus* | Korea | [25] |
| J | KU557671 | Donkey | *E. a. asinus* | China | [31] |
| J | KP262386 | Goat | *C. a. hircus* | China | [35] |
| J | KU685395 | Golden snub-nosed monkey | *R. roxellana* | China | [37] |
| J | AF135837 | Human | *H. sapiens* | China | [102] |
| J | KT267280 | Malayan sun bear | *Helarctos malayanus* | China | [6] |
| J | KT267297 | Meerkat | *Suricata suricatta* | China | [6] |
| J | MF693833 | Sambar deer | *R. unicolor* | Australia | This study |
| J | KR815512 | Sika deer | *C. nippon* | China | [107] |
| J | KJ867478 | White-tailed deer | *Odocoileus virginianus* | USA | [108] |
| J | AF135837 | Yak | *Bos grunniens* | China | [109] |
| J | KT267290 | Yak | *B. grunniens* | China | [6] |
| J | KT267278 | Zebra | *Equus burchellii* | China | [6] |
| J | AF135837 | Water | NA | China | [92] |
| Type IV | KT943975 | Helmeted guineafowl | *Numida meleagris* | Brazil | [1] |
| Type IV | KT943975 | Helmeted guineafowl | *N. meleagris* | Brazil | [8] |
| Type IV | AF242478 | Woodchuck | *Marmota monax* | USA | [75] |
| Type IV | KT750160 | Arctic fox | *V. lagopus* | China | [9] |
| Type IV | AF242478 | Black bear | *Ursus americanus* | USA | [75] |
| Type IV | AY371277 | Cattle | *B. taurus* | USA | [98] |
| Type IV (K) | DQ836343 | Cat | *F. catus* | Colombia | [110] |
| Type IV (K) | AB359945 | Cat | *F. catus* | Japan | [111] |
| Type IV | AF242478 | Cat | *F. catus* | China | [16] |
| Type IV | KF305582 | Cat | *F. catus* | China | [17] |
| Type IV (PtEb III) | DQ885579 | Cat | *F. catus* | Portugal | [29] |
| Type IV (K) | AF267141 | Cat | *F. catus* | Germany | [104] |
| Type IV (BEB5) | AY331009 | Cattle | *B. taurus* | Portugal | [112] |
| Type IV (BEB5) | AY331009 | Cattle | *B. taurus* | USA | [112] |
| Type IV (BEB5-Var) | AY331010 | Cattle | *B. taurus* | NA | NA |
| Type IV | AF242478 | Cynomolgus monkey | *M. fascicularis* | China | [113] |
| Type IV | KF305582 | Cynomolgus monkey | *M. fascicularis* | China | [28] |
| Type IV (CMITS1) | KT943975 | Dairy cow | *B. taurus* | Korea | [25] |
| Type IV (K) | EU650272 | Dog | *C. familiaris* | Colombia | [114] |
| Type IV | KJ668722 | Dog | *C. familiaris* | China | [15] |
| Type IV | AF242478 | Eastern chipmunk | *Tamias striatus* | USA | [75] |
| Type IV | AF242478 | Eastern gray squirrel | *Sciurus carolinensis* | USA | [75] |
| Type IV | AF242478 | Green monkey | *C. sabaeus* | China | [113] |
| Type IV | KJ728793 | Green monkey | *C. sabaeus* | China | [13] |
| Type IV | AF242478 | Human | *H. sapiens* | France | [115] |
| Type IV | AY371285 | Human | *H. sapiens* | Cameroon | [116] |
| Type IV | AF242478 | Human | *H. sapiens* | Nigeria | [117] |
| Type IV | AF242478 | Human | *H. sapiens* | Cameroon | [118] |
| Type IV | AF242478 | Human | *H. sapiens* | Nigeria | [119] |
| Type IV (K) | AF267141 | Human | *H. sapiens* | Uganda | [120] |
| Type IV (K) | DQ683756 | Human | *H. sapiens* | Gabon | [46] |
| Type IV (K) | DQ683752 | Human | *H. sapiens* | Cameroon | [46] |
| Type IV (K) | AF267141 | Human | *H. sapiens* | Niger | [73] |
| Type IV (K) | AF267141 | Human | *H. sapiens* | Malawi | [57] |
| Type IV (K) | AF267141 | Human | *H. sapiens* | Netherlands | [57] |
| Type IV (K) | AF267141 | Human | *H. sapiens* | Nigeria | [58] |
| Type IV | AY371285 | Human | *H. sapiens* | Nigeria | [59] |
| Type IV | AY371285 | Human | *H. sapiens* | Portugal | [66] |
| Type IV | AY371277 | Human | *H. sapiens* | Nigeria | [60] |
| Type IV | JQ029732 | Human | *H. sapiens* | China | [47] |
| Type IV (K) | AF267141 | Human | *H. sapiens* | Iran | [53] |
| Type IV | JX683798 | Human | *H. sapiens* | Nigeria | [61] |
| Type IV | JX683799 | Human | *H. sapiens* | Nigeria | [61] |
| Type IV | JX683800 | Human | *H. sapiens* | Nigeria | [61] |
| Type IV | JX683801 | Human | *H. sapiens* | Nigeria | [61] |
| Type IV | JX683803 | Human | *H. sapiens* | Nigeria | [61] |
| Type IV (K) | AF267141 | Human | *H. sapiens* | England | [51] |
| Type IV (Peru 2) | AY371277 | Human | *H. sapiens* | Peru | [62] |
| Type IV (Peru 2) | AY371277 | Human | *H. sapiens* | Peru | [63] |
| Type IV (Peru 2) | AY371277 | Human | *H. sapiens* | Peru | [64] |
| Type IV | AF242478 | Meadow vole | *Microtus pennsylvanicus* | USA | [75] |
| Type IV | KP057598 | Père David’s deer | *Elaphurus davidianus* | China | [121] |
| Type IV | KT267288 | Red fox | *V. vulpes* | China | [6] |
| Type IV | JX683801 | Rex rabbit | *O. cuniculus* | China | [85] |
| Type IV | AF242478 | Rhesus macaque | *M. mulatta* | China | [113] |
| Type IV | AY371277 | Rhesus macaque | *M. mulatta* | China | [122] |
| Type IV | KF305582 | Rhesus macaque | *M. mulatta* | China | [28] |
| Type IV | AF242478 | Ring-tailed lemur | *L. catta* | China | [113] |
| Type IV | KJ728793 | Ring-tailed lemur | *Lemur catta* | China | [13] |
| Type IV | MF693832 | Sambar deer | *R. unicolor* | Australia | This study |
| Type IV | AF242478 | Water | NA | China | [92] |
| Type IV | AY371277 | Water | NA | China | [123] |
| Type IV | AY371277 | Water | NA | Tunisia | [124] |
| Type IV | AF242478 | Water | NA | China | [94] |
| Type IV | AY371277 | Water | NA | China | [122] |
| Type IV (K) | AF267141 | Water | NA | Ireland | [124] |

Genotype BEB5-Var was reported in an unpublished study. NA = not available.

**References**

1. da Cunha MJR, Cury MC, Santín M. Widespread presence of human-pathogenic *Enterocytozoon bieneusi* genotypes in chickens. Vet Parasitol. 2016;217:108-12.

2. da Cunha MJR: **Diagnóstico e caracterização molecular de *Cryptosporidium* spp., *Giardia* spp. e *Enterocytozoon bieneusi* em aves das microrregiões de Uberlândia e Belo Horizonte, MG, Brasil**. 2017.

3. Zhao W, Yu S, Yang Z, Zhang Y, Zhang L, Wang R, et al. Genotyping of *Enterocytozoon bieneusi* (Microsporidia) isolated from various birds in China. Infect Genet Evol. 2016;40:151-4.

4. Müller MG, Kinne J, Schuster RK, Walochnik J. Outbreak of microsporidiosis caused by *Enterocytozoon bieneusi* in falcons. Vet Parasitol. 2008;152(1):67-78.

5. Pirestani M, Sadraei J, Forouzandeh M. Molecular characterization and genotyping of human related microsporidia in free-ranging and captive pigeons of Tehran, Iran. Infect Genet Evol. 2013;20:495-9.

6. Li J, Qi M, Chang Y, Wang R, Li T, Dong H, et al. Molecular characterization of *Cryptosporidium* spp., *Giardia duodenalis*, and *Enterocytozoon bieneusi* in captive wildlife at Zhengzhou Zoo, China. J Eukaryot Microbiol. 2015;62(6):833-9.

7. Perec-Matysiak A, Wesołowska M, Leśniańska K, Buńkowska-Gawlik K, Hildebrand J, Kicia M. Survey for zoonotic microsporidian pathogens in wild living urban rooks (*Corvus frugilegus*). J Eukaryot Microbiol. 2017.

8. da Cunha MJR, Cury MC, Santín M. Molecular identification of *Enterocytozoon bieneusi*, *Cryptosporidium*, and *Giardia* in Brazilian captive birds. Parasitol Res. 2017;116(2):487-93.

9. Zhang XX, Cong W, Lou ZL, Ma JG, Zheng WB, Yao QX, et al. Prevalence, risk factors and multilocus genotyping of *Enterocytozoon bieneusi* in farmed foxes (*Vulpes lagopus*), northern China. Parasit Vectors. 2016;9(1):72.

10. Zhao W, Zhang W, Yang Z, Liu A, Zhang L, Yang F, et al. Genotyping of *Enterocytozoon bieneusi* in farmed blue foxes (*Alopex lagopus*) and raccoon dogs (*Nyctereutes procyonoides*) in China. PLoS One. 2015;10(11):e0142611.

11. Perec-Matysiak A, Buńkowska-Gawlik K, Kváč M, Sak B, Hildebrand J, Leśniańska K. Diversity of *Enterocytozoon bieneusi* genotypes among small rodents in southwestern Poland. Vet Parasitol. 2015;214(3):242-6.

12. Li JQ, Dong HJ, Wang RJ, Yu FC, Wu YY, Chang YK, et al. An investigation of parasitic infections and review of molecular characterization of the intestinal protozoa in nonhuman primates in China from 2009 to 2015. Int J Parasitol Parasites Wildl. 2017;6(1):8-15.

13. Karim MR, Dong H, Li T, Yu F, Li D, Zhang L, et al. Predomination and new genotypes of *Enterocytozoon bieneusi* in captive nonhuman primates in zoos in China: high genetic diversity and zoonotic significance. PLoS One. 2015;10(2):e0117991.

14. Mynářová A, Foitová I, Kváč M, Květoňová D, Rost M, Morrogh-Bernard H, et al. Prevalence of *Cryptosporidium* spp., *Enterocytozoon bieneusi, Encephalitozoon* spp. and *Giardia intestinalis* in wild, semi-wild and captive Orangutans (*Pongo abelii and Pongo pygmaeus*) on Sumatra and Borneo, Indonesia. PLoS One. 2016;11(3):e0152771.

15. Karim MR, Dong H, Yu F, Jian F, Zhang L, Wang R, et al. Genetic diversity in *Enterocytozoon bieneusi* isolates from dogs and cats in China: host specificity and public health implications. J Clin Microbiol. 2014;52(9):3297-302.

16. Li W, Li Y, Song M, Lu Y, Yang J, Tao W, et al. Prevalence and genetic characteristics of *Cryptosporidium*, *Enterocytozoon bieneusi* and *Giardia duodenalis* in cats and dogs in Heilongjiang province, China. Vet Parasitol. 2015;208(3):125-34.

17. Xu H, Jin Y, Wu W, Li P, Wang L, Li N, et al. Genotypes of *Cryptosporidium* spp., *Enterocytozoon bieneusi* and *Giardia duodenalis* in dogs and cats in Shanghai, China. Parasit Vectors. 2016;9(1):121.

18. Mori H, Mahittikorn A, Thammasonthijarern N, Chaisiri K, Rojekittikhun W, Sukthana Y. Presence of zoonotic *Enterocytozoon bieneusi* in cats in a temple in central Thailand. Vet Parasitol. 2013;197(3):696-701.

19. Del Coco VF, Córdobaa MA, Bilbao G, de Almeida Castro P, Basualdo JA, Santín M. First report of *Enterocytozoon bieneusi* from dairy cattle in Argentina. Vet Parasitol. 2014;199(1):112-5.

20. da Silva Fiuza VR, Lopes CW, de Oliveira FC, Fayer R, Santín M. New findings of *Enterocytozoon bieneusi* in beef and dairy cattle in Brazil. Vet Parasitol. 2016;216:46-51.

21. Zhao W, Zhang W, Yang F, Zhang L, Wang R, Cao J, et al. *Enterocytozoon bieneusi* in dairy cattle in the northeast of China: genetic diversity of ITS Gene and evaluation of zoonotic transmission potential. J Eukaryot Microbiol. 2015;62(4):553-60.

22. Li J, Luo N, Wang C, Qi M, Cao J, Cui Z, et al. Occurrence, molecular characterization and predominant genotypes of *Enterocytozoon bieneusi* in dairy cattle in Henan and Ningxia, China. Parasit Vectors. 2016;9:142.

23. Qi M, Jing B, Jian FC, Wang RJ, Zhang SM, Wang HY, et al. Dominance of *Enterocytozoon bieneusi* genotype J in dairy calves in Xinjiang, northwest China. Parasitol Int. 2017;66(1):960-3.

24. Lee JH. Prevalence and molecular characteristics of *Enterocytozoon bieneusi* in cattle in Korea. Parasitol Res. 2007;101(2):391-6.

25. Lee JH. Molecular detection of *Enterocytozoon bieneusi* and identification of a potentially human-pathogenic genotype in milk. Appl Environ Microbiol. 2008;74(5):1664-6.

26. Abu Samra N, Thompson PN, Jori F, Zhang H, Xiao L. *Enterocytozoon bieneusi* at the wildlife/livestock interface of the Kruger National Park, South Africa. Vet Parasitol. 2012;190(3):587-90.

27. Ye J, Xiao L, Li J, Huang W, Amer SE, Guo Y, et al. Occurrence of human-pathogenic *Enterocytozoon bieneusi, Giardia duodenalis* and *Cryptosporidium* genotypes in laboratory macaques in Guangxi, China. Parasitol Int. 2014;63(1):132-7.

28. Karim MR, Wang R, Dong H, Zhang L, Li J, Zhang S, et al. Genetic polymorphism and zoonotic potential of *Enterocytozoon bieneusi* fom nonhuman primates in China. Appl Environ Microbiol. 2014;80(6):1893-8.

29. Lobo ML, Xiao L, Cama V, Stevens T, Antunes F, Matos O. Genotypes of *Enterocytozoon bieneusi* in mammals in Portugal. J Eukaryot Microbiol. 2006;53:61-4.

30. Zhang XX, Jiang J, Cai YN, Wang CF, Xu P, Yang GL, et al. Molecular characterization of *Enterocytozoon bieneusi* in domestic rabbits (*Oryctolagus cuniculus*) in northeastern China. Korean J Parasitol. 2016;54(1):81-5.

31. Yue DM, Ma JG, Li FC, Hou JL, Zheng WB, Zhao Q, et al. Occurrence of *Enterocytozoon bieneusi* in Donkeys (*Equus asinus*) in China: A Public Health Concern. Front Microbiol. 2017;8:565.

32. Sak B, Kváč M, Hanzlíková D, Albrecht T, Piálek J. The first report on natural *Enterocytozoon bieneusi* and *Encephalitozoon* spp. infections in wild East-European House Mice (*Mus musculus musculus*) and West-European House Mice (*M. m. domesticus*) in a hybrid zone across the Czech Republic-Germany border. Vet Parasitol. 2011;178(3):246-50.

33. Li W, Deng L, Wu K, Huang X, Song Y, Su H, et al. Presence of zoonotic *Cryptosporidium scrofarum*, *Giardia duodenalis* assemblage A and *Enterocytozoon bieneusi* genotypes in captive Eurasian wild boars (*Sus scrofa*) in China: potential for zoonotic transmission. Parasit Vectors. 2017;10(1):10.

34. Zhao W, Zhang W, Yang D, Zhang L, Wang R, Liu A. Prevalence of *Enterocytozoon bieneusi* and genetic diversity of ITS genotypes in sheep and goats in China. Infect Genet Evol. 2015;32:265-70.

35. Shi K, Li M, Wang X, Li J, Karim MR, Wang R, et al. Molecular survey of *Enterocytozoon bieneusi* in sheep and goats in China. Parasit Vectors. 2016;9(1):23.

36. Du SZ, Zhao GH, Shao JF, Fang YQ, Tian GR, Zhang LX, et al. *Cryptosporidium spp., Giardia intestinalis,* and *Enterocytozoon bieneusi* in captive non-human primates in Qinling mountains. Korean J Parasitol. 2015;53(4):395-402.

37. Yu F, Wu Y, Li T, Cao J, Wang J, Hu S, et al. High prevalence of *Enterocytozoon bieneusi* zoonotic genotype D in captive golden snub-nosed monkey (*Rhinopithecus roxellanae)* in zoos in China. BMC Vet Res. 2017;13(1):158.

38. Li W, Deng L, Yu X, Zhong Z, Wang Q, Liu X, et al. Multilocus genotypes and broad host-range of *Enterocytozoon bieneusi* in captive wildlife at zoological gardens in China. Parasit Vectors. 2016;9(1):395.

39. Zhao GH, Du SZ, Wang HB, Hu XF, Deng MJ, Yu SK, et al. First report of zoonotic *Cryptosporidium* spp., *Giardia intestinalis* and *Enterocytozoon bieneusi* in golden takins (*Budorcas taxicolor bedfordi)*. Infect Genet Evol. 2015;34:394-401.

40. Sak B, Petrzelkova KJ, Kvetonova D, Mynarova A, Shutt KA, Pomajbikova K, et al. Long-term monitoring of *microsporidia*, *Cryptosporidium* and *Giardia* infections in western Lowland Gorillas (*Gorilla gorilla gorilla*) at different stages of habituation in Dzanga Sangha Protected Areas, Central African Republic. PLoS One. 2013;8(8):e71840.

41. Qi M, Wang R, Wang H, Jian F, Li J, Zhao J, et al. *Enterocytozoon bieneusi* genotypes in grazing horses in China and their zoonotic transmission potential. J Eukaryot Microbiol. 2016;63(5):591-7.

42. Deng L, Li W, Zhong Z, Gong C, Liu X, Huang X, et al. Molecular characterization and multilocus genotypes of *Enterocytozoon bieneusi* among horses in southwestern China. Parasit Vectors. 2016;9(1):561.

43. Santín M, Vecino JAC, Fayer R. A zoonotic genotype of *Enterocytozoon bieneusi* in horses. J Parasitol. 2010;96(1):157-61.

44. Wagnerová P, Sak B, Květoňová D, Buňatová Z, Civišová H, Maršálek M, et al. *Enterocytozoon bieneusi* and *Encephalitozoon cuniculi* in horses kept under different management systems in the Czech Republic. Vet Parasitol. 2012;190(3):573-7.

45. Feng Y, Li N, Dearen T, Lobo ML, Matos O, Cama V, et al. Development of a multilocus sequence typing tool for high-resolution genotyping of *Enterocytozoon bieneusi*. Appl Environ Microbiol. 2011;77(14):4822-8.

46. Breton J, Bart-Delabesse E, Biligui S, Carbone A, Seiller X, Okome-Nkoumou M, et al. New highly divergent rRNA sequence among biodiverse genotypes of *Enterocytozoon bieneusi* strains isolated from humans in Gabon and Cameroon. J Clin Microbiol. 2007;45(8):2580-9.

47. Wang L, Zhang H, Zhao X, Zhang L, Zhang G, Guo M, et al. Zoonotic *Cryptosporidium species* and *Enterocytozoon bieneusi* genotypes in HIV-positive patients on antiretroviral therapy. J Clin Microbiol. 2013;51(2):557-63.

48. Wang L, Xiao L, Duan L, Ye J, Guo Y, Guo M, et al. Concurrent infections of *Giardia duodenalis, Enterocytozoon bieneusi,* and *Clostridium difficile* in children during a cryptosporidiosis outbreak in a pediatric hospital in China. PLoS Negl Trop Dis. 2013;. 7(9):e2437.

49. Wang T, Fan Y, Koehler AV, Ma G, Li T, Hu M, et al. First survey of *Cryptosporidium*, *Giardia* and *Enterocytozoon* in diarrhoeic children from Wuhan, China. Infect Genet Evol. 2017;51:127-31.

50. Kicia M, Wesolowska M, Jakuszko K, Kopacz Z, Sak B, Květonová D, et al. Concurrent infection of the urinary tract with *Encephalitozoon cuniculi* and *Enterocytozoon bieneusi* in a renal transplant recipient. J Clin Microbiol. 2014;52(5):1780-2.

51. Sadler F, Peake N, Borrow R, Rowl PL, Wilkins EG, Curry A. Genotyping of *Enterocytozoon bieneusi* in AIDS Patients from the north west of England. J Infect. 2002;44(1):39-42.

52. Li W, Cama V, Akinbo FO, Ganguly S, Kiulia NM, Zhang X, et al. Multilocus sequence typing of *Enterocytozoon bieneusi*: lack of geographic segregation and existence of genetically isolated sub-populations. Infect Genet Evol. 2013;14:111-9.

53. Agholi M, Hatam GR, Motazedian MH. HIV/AIDS-associated opportunistic protozoal diarrhea. AIDS Res Hum Retroviruses. 2013;29(1):35-41.

54. Mirjalali H, Mirhendi H, Meamar AR, Mohebali M, Askari Z, Mirsamadi ES, et al. Genotyping and molecular analysis of *Enterocytozoon bieneusi* isolated from immunocompromised patients in Iran. Infect Genet Evol. 2015;36:244-9.

55. Kazemi E, Tavalla M, Maraghi S, Yad MJ, Latifi M. Frequency of microsporidial infection in immunocompromised patients with staining and molecular methods based on internal transcribed spacer region gene in two cities of southwest Iran during 2013-2014. Asian J Pharm Res Health Care. 2016;9(1):7-16.

56. Tavalla M, Mardani-Kateki M, Abdizadeh R, Nashibi R, Rafie A, Khademvatan S. Molecular identification of *Enterocytozoon bieneusi* and *Encephalitozoon* spp. in immunodeficient patients in Ahvaz, southwest of Iran. Acta Trop. 2017.

57. ten Hove RJ, Van Lieshout L, Beadsworth MB, Perez MA, Spee K, Claas EC, et al. Characterization of genotypes of *Enterocytozoon bieneusi* in immunosuppressed and immunocompetent patient groups. J Eukaryot Microbiol. 2009;56(4):388-93.

58. Ayinmode AB, Ojuromi OT, Xiao L. Molecular Identification of *Enterocytozoon bieneusi* Isolates from Nigerian Children. J Parasitol Res. 2011;2011.

59. Akinbo FO, Okaka CE, Omoregie R, Dearen T, Leon ET, Xiao L. Molecular epidemiologic characterization of *Enterocytozoon bieneusi* in HIV-infected persons in Benin City, Nigeria. Am J Trop Med Hyg. 2012;86(3):441-5.

60. Maikai BV, Umoh JU, Lawal IA, Kudi AC, Ejembi CL, Xiao L. Molecular characterizations of *Cryptosporidium*, *Giardia*, and *Enterocytozoon* in humans in Kaduna State, Nigeria. Exp Parasitol. 2012;131(4):452-6.

61. Ayinmode AB, Zhang H, Dada-Adegbola HO, Xiao L. *Cryptosporidium hominis* subtypes and *Enterocytozoon bieneusi* genotypes in HIV-infected persons in Ibadan, Nigeria. Zoonoses Public Health. 2014;61(4):297-303.

62. Sulaiman IM, Bern C, Gilman R, Cama V, Kawai V, Vargas D, et al. A molecular biologic study of *Enterocytozoon bieneusi* in HIV-infected patients in Lima, Peru. J Eukaryot Microbiol. 2003;50 Suppl:591-6.

63. Bern C, Kawai V, Vargas D, Rabke-Verani J, Williamson J, Chavez-Valdez R, et al. The epidemiology of intestinal microsporidiosis in patients with HIV/AIDS in Lima, Peru. J Infect Dis. 2005;191(10):1658-64.

64. Cama VA, Pearson J, Cabrera L, Pacheco L, Gilman R, Meyer S, et al. Transmission of *Enterocytozoon bieneusi* between a child and guinea pigs. J Clin Microbiol. 2007;45(8):2708-10.

65. Kicia M, Wesolowska M, Kopacz Z, Jakuszko K, Sak B, Květonová D, et al. Prevalence and molecular characteristics of urinary and intestinal microsporidia infections in renal transplant recipients. Clin Microbiol Infect. 2016;22(5):462 e5-9.

66. Lobo ML, Xiao L, Antunes F, Matos O. Microsporidia as emerging pathogens and the implication for public health: a 10-year study on HIV-positive and -negative patients. Int J Parasitol. 2012;42(2):197-205.

67. Sokolova OI, Demyanov AV, Bowers LC, Didier ES, Yakovlev AV, Skarlato SO, et al. Emerging microsporidian infections in Russian HIV-infected patients. J Clin Microbiol. 2011;49(6):2102-8.

68. Leelayoova S, Subrungruang I, Suputtamongkol Y, Worapong J, Petmitr PC, Mungthin M. Identification of genotypes of *Enterocytozoon bieneusi* from stool samples from human immunodeficiency virus-infected patients in Thailand. J Clin Microbiol. 2006;44(8):3001-4.

69. Saksirisampant W, Prownebon J, Saksirisampant P, Mungthin M, Siripatanapipong S, Leelayoova S. Intestinal parasitic infections: prevalences in HIV/AIDS patients in a Thai AIDS-care centre. Ann Trop Med Parasitol. 2009;103(7):573-81.

70. Mori H, Mahittikorn A, Watthanakulpanich D, Komalamisra C, Sukthana Y. Zoonotic potential of *Enterocytozoon bieneusi* among children in rural communities in Thailand. Parasite. 2013;20:14.

71. Prasertbun R, Mori H, Pintong AR, Sanyanusin S, Popruk S, Komalamisra C, et al. Zoonotic potential of *Enterocytozoon* genotypes in humans and pigs in Thailand. Vet Parasitol. 2017;233:73-9.

72. Chabchoub N, Abdelmalek R, Breton J, Kanoun F, Thellier M, Bouratbine A, et al. Genotype identification of *Enterocytozoon bieneusi* isolates from stool samples of HIV-infected Tunisian patients. Parasite. 2012;19(2):147-51.

73. Espern A, Morio F, Miegeville M, Illa H, Abdoulaye M, Meyssonnier V, et al. Molecular study of microsporidiosis due to *Enterocytozoon bieneusi* and *Encephalitozoon intestinalis* among human immunodeficiency virus-infected patients from two geographical areas: Niamey, Niger, and Hanoi, Vietnam. J Clin Microbiol. 2007;45(9):2999-3002.

74. Sulaiman IM, Fayer R, Lal AA, Trout JM, Schaefer FW, Xiao L. Molecular characterization of microsporidia indicates that wild mammals harbor host-sdapted *Enterocytozoon* spp. as well as human-pathogenic *Enterocytozoon bieneusi*. Appl Environ Microbiol. 2003;69(8):4495-501.

75. Guo Y, Alderisio KA, Yang W, Cama V, Feng Y, Xiao L. Host specificity and source of *Enterocytozoon bieneusi* genotypes in a drinking source watershed. Appl Environ Microbiol. 2014;80(1):218-25.

76. Zhong Z, Li W, Deng L, Song Y, Wu K, Tian Y, et al. Multilocus genotyping of *Enterocytozoon bieneusi* derived from nonhuman primates in southwest China. PLoS One. 2017;12(5):e0176926.

77. Li W, Kiulia NM, Mwenda JM, Nyachieo A, Taylor MB, Zhang X, et al. *Cyclospora papionis*, *Cryptosporidium hominis*, and human-pathogenic *Enterocytozoon bieneusi* in captive baboons in Kenya. J Clin Microbiol. 2011;49(12):4326-9.

78. Li W, Diao R, Yang J, Xiao L, Lu Y, Li Y, et al. High diversity of human-pathogenic *Enterocytozoon bieneusi* genotypes in swine in northeast China. Parasitol Res. 2014;113(3):1147-53.

79. Zhao W, Zhang W, Yang F, Cao J, Liu H, Yang D, et al. High prevalence of *Enterocytozoon bieneusi* in asymptomatic pigs and assessment of zoonotic risk at the genotype level. Appl Environ Microbiol. 2014;80(12):3699-707.

80. Sak B, Kváč M, Hanzlíková D, Cama V. First report of *Enterocytozoon bieneusi* infection on a pig farm in the Czech Republic. Vet Parasitol. 2008;153(3):220-4.

81. Abe N, Kimata I. Molecular survey of *Enterocytozoon bieneusi* in a Japanese porcine population. Vector Borne Zoonotic Dis. 2010;10(4):425-7.

82. Yang Y, Lin Y, Li Q, Zhang S, Tao W, Wan Q, et al. Widespread presence of human-pathogenic *Enterocytozoon bieneusi* genotype D in farmed foxes (*Vulpes vulpes*) and raccoon dogs (*Nyctereutes procyonoides*) in China: first identification and zoonotic concern. Parasitol Res. 2015;114(11):4341-8.

83. Xu C, Ma X, Zhang H, Zhang XX, Zhao JP, Ba HX, et al. Prevalence, risk factors and molecular characterization of *Enterocytozoon bieneusi* in raccoon dogs (*Nyctereutes procyonoides*) in five provinces of Northern China. Acta Trop. 2016;161:68-72.

84. Deng L, Li W, Yu X, Gong C, Liu X, Zhong Z, et al. First report of the human-pathogenic *Enterocytozoon bieneusi* from Red-Bellied Tree Squirrels (*Callosciurus erythraeus*) in Sichuan, China. PLoS One. 2016;11(9):e0163605.

85. Yang Z, Zhao W, Shen Y, Zhang W, Shi Y, Ren G, et al. Subtyping of *Cryptosporidium cuniculus* and genotyping of *Enterocytozoon bieneusi* in rabbits in two farms in Heilongjiang Province, China. Parasite. 2016;23:52.

86. Chalifoux LV, Carville A, Pauley D, Thompson B, Lackner AA, Mansfield KG. *Enterocytozoon bieneusi* as a cause of proliferative serositis in simian immunodeficiency virus-infected immunodeficient macaques (*Macaca mulatta*). Arch Pathol Lab Med. 2000;124(10):1480-4.

87. Huang J, Zhang Z, Yang Y, Wang R, Zhao J, Jian F, et al. New genotypes of *Enterocytozoon bieneusi* isolated from sika deer and red deer in China. Front Microbiol. 2017;8:879.

88. Buckholt MA, Lee JH, Tzipori S. Prevalence of *Enterocytozoon bieneusi* in swine: an 18-month survey at a slaughterhouse in Massachusetts. Appl Environ Microbiol. 2002;68(5):2595-9.

89. Němejc K, Sak B, Květoňová D, Hanzal V, Janiszewski P, Forejtek P, et al. Prevalence and diversity of *Encephalitozoon* spp. and *Enterocytozoon bieneusi* in wild boars (*Sus scrofa*) in Central Europe. Parasitol Res. 2014;113(2):761-7.

90. Hu Y, Feng Y, Huang C, Xiao L. Occurrence, source, and human infection potential of *Cryptosporidium* and E*nterocytozoon bieneusi* in drinking source water in Shanghai, China, during a pig carcass disposal incident. Environ Sci Technol. 2014;48(24):14219-27.

91. Ma J, Feng Y, Hu Y, Villegas EN, Xiao L. Human infective potential of *Cryptosporidium* spp., *Giardia duodenalis* and *Enterocytozoon bieneusi* in urban wastewater treatment plant effluents. J Water Health. 2016;14(3):411-23.

92. Ye J, Yan J, Xu J, Ma K, Yang X. Zoonotic *Enterocytozoon bieneusi* in raw wastewater in Zhengzhou, China. Folia Parasitol (Praha). 2017;64:002.

93. Galván AL, Magnet A, Izquierdo F, Fenoy S, Rueda C, Fernandez Vadillo C, et al. Molecular characterization of human-pathogenic microsporidia and *Cyclospora cayetanensis* isolated from various water sources in Spain: a year-long longitudinal study. Appl Environ Microbiol. 2013;79(2):449-59.

94. Huang C, Hu Y, Wang L, Wang Y, Li N, Guo Y, et al. Environmental transport of emerging human-pathogenic *Cryptosporidium* species and subtypes through combined sewer overflow and wastewater. Appl Environ Microbiol. 2017;AEM-00682.

95. Reetz J, Rinder H, Thomschke A, Manke H, Schwebs M, Bruderek A. First detection of the microsporidium *Enterocytozoon bieneusi* in non-mammalian hosts (chickens). Int J Parasitol. 2002;32(7):785-7.

96. Santín M, Fayer R. A longitudinal study of *Enterocytozoon bieneusi* in dairy cattle. Parasitol Res. 2009;105(1):141-4.

97. Wang XT, Wang RJ, Ren GJ, Yu ZQ, Zhang LX, Zhang SY, et al. Multilocus genotyping of *Giardia* duodenalis and *Enterocytozoon bieneusi* in dairy and native beef (Qinchuan) calves in Shaanxi province, northwestern China. Parasitol Res. 2016;115(3):1355-61.

98. Santín M, Dargatz D, Fayer R. Prevalence and genotypes of *Enterocytozoon bieneusi* in weaned beef calves on cow-calf operations in the USA. Parasitol Res. 2012;110(5):2033-41.

99. Rinder H, Thomschke A, Dengjel B, Gothe R, Löscher T, Zahler M. Close genotypic relationship between *Enterocytozoon bieneusi* from humans and pigs and first fetection in cattle. J Parasitol. 2000;86(1):185-8.

100.Baroudi D, Khelef D, Hakem A, Abdelaziz A, Chen X, Lysen C, et al. Molecular characterization of zoonotic pathogens *Cryptosporidium* spp., *Giardia duodenalis* and *Enterocytozoon bieneusi* in calves in Algeria. Vet Parasitol. 2017;8:66-9.

101.Jiang Y, Tao W, Wan Q, Li Q, Yang Y, Lin Y, et al. Zoonotic and potentially host-adapted *Enterocytozoon bieneusi* genotypes in sheep and cattle in northeast China and an increasing concern about the zoonotic Importance of previously considered ruminant-adapted genotypes. Appl Environ Microbiol. 2015;81(10):3326-35.

102.Zhang X, Wang Z, Su Y, Liang X, Sun X, Peng S, et al. Identification and genotyping of *Enterocytozoon bieneusi* in China. J Clin Microbiol. 2011;49(5):2006-8.

103.Ma J, Li P, Zhao X, Xu H, Wu W, Wang Y, et al. Occurrence and molecular characterization of *Cryptosporidium* spp. and *Enterocytozoon bieneusi* in dairy cattle, beef cattle and water buffaloes in China. Vet Parasitol. 2015;207(3):220-7.

104.Dengjel B, Zahler M, Hermanns W, Heinritzi K, Spillmann T, Thomschke A, et al. Zoonotic potential of *Enterocytozoon bieneusi*. J Clin Microbiol. 2001;39(12):4495-9.

105. Fayer R, Santín M, Trout JM. *Enterocytozoon bieneusi* in mature dairy cattle on farms in the eastern United States. Parasitol Res. 2007;102(1):15-20.

106.Santín M, Trout JM, Fayer R. *Enterocytozoon bieneusi* genotypes in dairy cattle in the eastern United States. Parasitol Res. 2005;97(6):535-8.

107.Zhang XX, Cong W, Liu GH, Ni XT, Ma JG, Zheng WB, et al. Prevalence and genotypes of *Enterocytozoon bieneusi* in sika deer in Jilin province, northeastern China. Acta Parasitol. 2016;61(2):382-8.

108.Santín M, Fayer R. *Enterocytozoon bieneusi, Giardia,* and *Cryptosporidium* infecting white-tailed deer. J Eukaryot Microbiol. 2015;62(1):34-43.

109.Ma J, Cai J, Ma J, Feng Y, Xiao L. *Enterocytozoon bieneusi* genotypes in yaks (*Bos grunniens*) and their public health potential. J Eukaryot Microbiol. 2015;62(1):21-5.

110. Santín M, Trout JM, Vecino JA, Dubey JP, Fayer R. *Cryptosporidium*, *Giardia* and *Enterocytozoon bieneusi* in cats from Bogota (Colombia) and genotyping of isolates. Vet Parasitol. 2006;141(3):334-9.

111. Abe N, Kimata I, Iseki M. Molecular evidence of *Enterocytozoon bieneus*i in Japan. J Vet Med Sci. 2009;71(2):217-9.

112. Sulaiman IM, Fayer R, Yang C, Santín M, Matos O, Xiao L. Molecular characterization of *Enterocytozoon bieneusi* in cattle indicates that only some isolates have zoonotic potential. Parasitol Res. 2004;92(4):328-34.

113. Li J, Dong H, Wang R, Yu F, Wu Y, Chang Y, et al. An investigation of parasitic infections and review of molecular characterization of the intestinal protozoa in nonhuman primates in China from 2009 to 2015. Int J Parasitol Parasites Wildl. 2017;6(1):8-15.

114. Santín M, Cortes Vecino JA, Fayer R. *Enterocytozoon bieneusi* genotypes in dogs in Bogota, Colombia. Am J Trop Med Hyg. 2008;79(2):215-7.

115. Liguory O, Sarfati C, Derouin F, Molina JM. Evidence of different *Enterocytozoon bieneusi* genotypes in patients with and without human immunodeficiency virus infection. J Clin Microbiol. 2001;39(7):2672-4.

116. Sarfati C, Bourgeois A, Menotti J, Liegeois F, Moyou-Somo R, Delaporte E, et al. Prevalence of intestinal parasites including microsporidia in human immunodeficiency virus-infected adults in Cameroon: a cross-sectional study. Am J Trop Med Hyg. 2006;74(1):162-4.

117. Akinbo FO, Okaka CE, Omoregie R, Adamu H, Xiao L. Unusual *Enterocytozoon bieneusi* genotypes and *Cryptosporidium* hominis subtypes in HIV-infected patients on highly active antiretroviral therapy. Am J Trop Med Hyg. 2013;89(1):157-61.

118. Ndzi ES, Asonganyi T, Nkinin MB, Xiao L, Didier ES, Bowers LC, et al. Fast technology analysis enables identification of species and genotypes of latent microsporidia infections in healthy native cameroonians. J Eukaryot Microbiol. 2016;63(2):146-52.

119. Ojuromi OT, Duan L, Izquierdo F, Fenoy SM, Oyibo WA, Del Aguila C, et al. Genotypes of *Cryptosporidium* spp. and *Enterocytozoon bieneusi* in human immunodeficiency virus-infected patients in Lagos, Nigeria. J Eukaryot Microbiol. 2016;63(4):414-8.

120.Tumwine JK, Kekitiinwa A, Nabukeera N, Akiyoshi DE, Buckholt MA, Tzipori S. *Enterocytozoon bieneusi* among children with diarrhea attending Mulago Hospital in Uganda. Am J Trop Med Hyg. 2002;67(3):299-303.

121.Zhang Z, Huang J, Karim MR, Zhao J, Dong H, Ai W, et al. Zoonotic *Enterocytozoon bieneusi* genotypes in Père David's deer (*Elaphurus davidianus*) in Henan, China. Exp Parasitol. 2015;155:46-8.

122.Ye J, Xiao L, Ma J, Guo M, Liu L, Feng Y. Anthroponotic enteric parasites in monkeys in public park, China. Emerg Infect Dis. 2012;18(10):1640-3.

123.Li N, Xiao L, Wang L, Zhao S, Zhao X, Duan L, et al. Molecular surveillance of *Cryptosporidium* spp., *Giardia duodenalis*, and *Enterocytozoon bieneusi* by genotyping and subtyping parasites in wastewater. PLoS Negl Trop Dis. 2012;6(9):e1809.

124. Graczyk TK, Lucy FE, Mashinsky Y, Andrew Thompson RC, Koru O, daSilva AJ. Human zoonotic enteropathogens in a constructed free-surface flow wetland. Parasitol Res. 2009;105(2):423-8.
